# Meta-organism gene expression reveals that the impact of nitrate enrichment on coral larvae is mediated by their associated Symbiodiniaceae and prokaryotic assemblages

**DOI:** 10.1186/s40168-023-01495-0

**Published:** 2023-04-26

**Authors:** Haoya Tong, Fang Zhang, Jin Sun, Shelby E. McIlroy, Weipeng Zhang, Yan Wang, Hui Huang, Guowei Zhou, Pei-Yuan Qian

**Affiliations:** 1 Southern Marine Science and Engineering Guangdong Laboratory (Guangzhou), Nansha, Guangzhou, China; 2 Department of Ocean Science, The Hong Kong University of Science and Technology, Hong Kong SAR, China; 3 CAS Key Laboratory of Tropical Marine Bio-Resources and Ecology, South China Sea Institute of Oceanology, Institute of South China Sea Ecology and Environmental Engineering, Chinese Academy of Sciences, Guangzhou, China; 4 CAS-HKUST Sanya Joint Laboratory of Marine Science Research and Hainan Key Laboratory of Tropical Marine Biotechnology, Tropical Marine Biological Research Station in Hainan, Chinese Academy of Sciences, Sanya, China; 5 Institute of Evolution and Marine Biodiversity, Ocean University of China, Qingdao, China; 6 The Swire Institute of Marine Science, School of Biological Sciences, The University of Hong Kong, Hong Kong SAR, China; 7 College of Marine Life Sciences, Ocean University of China, Qingdao, China

**Keywords:** Coral larvae, Coral meta-organism associations, Coral reef microbiome, Nitrogen pollution

## Abstract

**Background:**

Coral meta-organisms consist of the coral, and its associated Symbiodiniaceae (dinoflagellate algae), bacteria, and other microbes. Corals can acquire photosynthates from Symbiodiniaceae, whilst Symbiodiniaceae uses metabolites from corals. Prokaryotic microbes provide Symbiodiniaceae with nutrients and support the resilience of corals as meta-organisms. Eutrophication is a major cause of coral reef degradation; however, its effects on the transcriptomic response of coral meta-organisms remain unclear, particularly for prokaryotic microbes associated with corals in the larval stage. To understand acclimation of the coral meta-organism to elevated nitrate conditions, we analyzed the physiological and transcriptomic responses of *Pocillopora damicornis* larvae, an ecologically important scleractinian coral, after 5 days of exposure to elevated nitrate levels (5, 10, 20, and 40 µM).

**Results:**

The major differentially expressed transcripts in coral, Symbiodiniaceae, and prokaryotic microbes included those related to development, stress response, and transport. The development of Symbiodiniaceae was not affected in the 5 and 20 µM groups but was downregulated in the 10 and 40 µM groups. In contrast, prokaryotic microbe development was upregulated in the 10 and 40 µM groups and downregulated in the 5 and 20 µM groups. Meanwhile, coral larval development was less downregulated in the 10 and 40 µM groups than in the 5 and 20 µM groups. In addition, multiple larval, Symbiodiniaceae, and prokaryotic transcripts were significantly correlated with each other. The core transcripts in correlation networks were related to development, nutrient metabolism, and transport. A generalized linear mixed model, using least absolute shrinkage and selection operator, demonstrated that the Symbiodiniaceae could both benefit and cost coral larval development. Furthermore, the most significantly correlated prokaryotic transcripts maintained negative correlations with the physiological functions of Symbiodiniaceae.

**Conclusions:**

Results suggested that Symbiodiniaceae tended to retain more nutrients under elevated nitrate concentrations, thereby shifting the coral-algal association from mutualism towards parasitism. Prokaryotic microbes provided Symbiodiniaceae with essential nutrients and may control Symbiodiniaceae growth through competition, whereby prokaryotes can also restore coral larval development inhibited by Symbiodiniaceae overgrowth.

Video Abstract

**Supplementary Information:**

The online version contains supplementary material available at 10.1186/s40168-023-01495-0.

## Background

Coral reefs support high biodiversity and provide abundant natural resources; however, they have rapidly declined in recent years owing to anthropogenic impacts, including global climate change, pollution from coastal cities, overfishing, and other human activities [1, 2]. Excessive nitrate from anthropogenic activities is one of the main stressors leading to coastal coral reef degradation by reducing coral resistance to environmental stress, reproduction, and recruitment, as well as increasing mass mortality [3–6]. High nitrate concentrations impair coral growth and calcification, whilst ammonia has lesser effects [7]. Meanwhile, enriched nutrients which maintained N to P ratios were less damaging to coral health [8–10]. However, some evidence showed that nitrogen with phosphate enrichment can lead to parasitism by the symbiotic algae, Symbiodiniaceae, within the coral meta-organism [11, 12]. Despite various studies on coral-algal associations under nutrient enrichments, little is known about the roles of prokaryotic microbes within the coral meta-organisms under eutrophic conditions [13] even though they are known for maintaining critical functions in coral meta-organisms, including nitrogen and carbon metabolism [14, 15].

The coral as a meta-organism consists of the coral and associated Symbiodiniaceae, bacteria, archaea, and other microbes which are fundamental in creating a thriving coral reef ecosystem within an oligotrophic ocean [16, 17]. Corals acquire photosynthates from Symbiodiniaceae, whereas Symbiodiniaceae and prokaryotic microbes gain access to coral metabolites [18–20]. Symbiodiniaceae are known to provide the majority of a coral’s energetic needs through photosynthesis and the assimilation of dissolved inorganic nitrogen [21]. Meanwhile, various coral associated prokaryotic microbes also maintain genetic machinery for nitrogen cycling and may also provide important contributions to nitrogen cycling within the coral meta-organisms. These intimate relationships result in complex coral responses to nutrient pollution.

Several mechanisms have been proposed to explain the decline in coral cover under nutrient pollution. One common theory is that the mechanism of decline is caused by macroalgal overgrowth [22–24], which causes poor light penetration and results in an anoxic environment that ultimately suffocates the coral [25–27]. The dissolved organic carbon (DOC), disease, algae, and microorganisms (DDAM) model predicts that increasing DOC caused by macroalgal overgrowth leads to heterotrophic microbial growth and coral disease [28]. Another widely accepted hypothesis is that imbalanced nutrients destabilize the coral-algal symbiosis, making coral vulnerable under thermal stress [29]. Symbiodiniaceae could retain more photosynthates under high nitrogen concentrations [30], which may limit CO_2_ and eventually cause coral bleaching. Furthermore, even though corals maintain Symbiodiniaceae in a nitrogen-limited internal environment, high nitrogen concentrations followed by elevated nitrate assimilation in coral meta-organisms lead to phosphate starvation and increased cell division rates [31]. In turn, high nitrogen concentrations can affect Symbiodiniaceae susceptibility to thermal stress and thus impair the coral-algal symbiosis [29]. However, the associations between nitrate concentration and Symbiodiniaceae growth are not completely known. Some studies also found that high nitrate concentrations inhibited Symbiodiniaceae density in corals [7]. In addition, nitrogen concentration affects the photosynthetic efficiency of Symbiodiniaceae and thus may affect the coral-algal symbiosis [4].

The mechanisms by which coral-associated prokaryotic microbes may mediate the acclimation of coral meta-organisms under nutrient stress are poorly understood, particularly at the coral larval stage [13]. However, various contradictory findings have been reported: some studies found that the prokaryotic community shifted into a dysbiotic state because of the limited DOC available under high N to P ratios [32], whilst some demonstrated an inflexible prokaryotic community under nutrient stress [33]. How prokaryotic microbial functions are altered under nutrient stress has been barely explored.

The dispersal and recruitment of coral larvae play critical roles in establishing coral reefs [34, 35]; however, the effect of eutrophication on coral larvae is still inconclusive. Coral larval performance under nutrient stress depends on coral species, nutrient identity (such as nitrate and ammonia) [7], N to P ratio [9], and nutrient ecological source. High nitrate concentrations can impair coral larval survivorship, particularly coral species with horizontally transmitted Symbiodiniaceae [36]. However, *Porites astreoides* larvae were found to have increased settlement rates under nitrate enrichment [37] whereas elevated ammonia decreased the settlement of *Diploria strigosa* [38]. Enriched nutrients with a balanced N to P ratio increased *Montipora capitata* larval size but reduced *Lobactis scutaria* larval size [36]. Besides species-specific effects, larval nutrient sensitivity is also related to life-history traits of corals, including reproduction and symbiont transmission mode [36]. Furthermore, the context of the environment, where corals are originally from, can affect a coral’s responses to nutrient stress. Despite their critical role in nutrient cycling, however, the associations amongst coral larval meta-organisms under nutrient stress, particularly coral- and algal-prokaryotic relationships, remain largely unknown.

*Pocillopora damicornis* is a hermaphroditic brooder, and it is one of the most widespread corals in the world [34, 39]. In the present study, *P. damicornis* larvae were exposed to four different nitrate concentrations including ambient seawater as the control. Therefore, photophysiology and transcriptome changes in coral meta-organisms were examined to explore the potential mechanism of acclimation, particularly the role of associations amongst coral meta-organisms under eutrophic conditions.

## Results

### De novo assembly of reference transcriptomes for coral, Symbiodiniaceae, and prokaryotic microbes

A reference transcriptome from a total of 122 GB of clean reads was generated from all samples under all treatments and the control with 47,805,710 ± 3,779,866 pair reads per sample (mean ± SD). A total of 677,207 transcripts were assembled, with a sample alignment of over 98%. In addition, the statistics based on assembled transcripts and the longest isoform per gene including N50, median, and average contig length, are listed in Table S1 of Additional File 1. After blast to respective databases, 335,941 and 169,031 transcripts were identified as a primary reference transcriptome construction for coral and Symbiodiniaceae, respectively. Kraken2 detected 68,020 prokaryotic transcripts to construct a primary reference transcriptome for prokaryotes. Samples had around 27.1, 32.8, and 2.6 million read pairs on average for mapping primary coral, Symbiodiniaceae, and prokaryotic reference transcriptome, respectively. After removing shared transcripts among different reference transcriptomes, a total of 110,546 coral transcripts, 35,044 Symbiodiniaceae transcripts, and 33,122 prokaryotic transcripts were retained for generating final reference transcriptomes for downstream analysis. Detailed transcript statistics of each sample for mapping primary reference transcriptome are shown in Table S2, Additional File 1. The coral reference transcriptome displayed a BUSCO transcriptome completeness of 89%.

### Differentially expressed transcripts (DETs) in coral, Symbiodiniaceae, and prokaryotic microbes

**Fig. 1:**
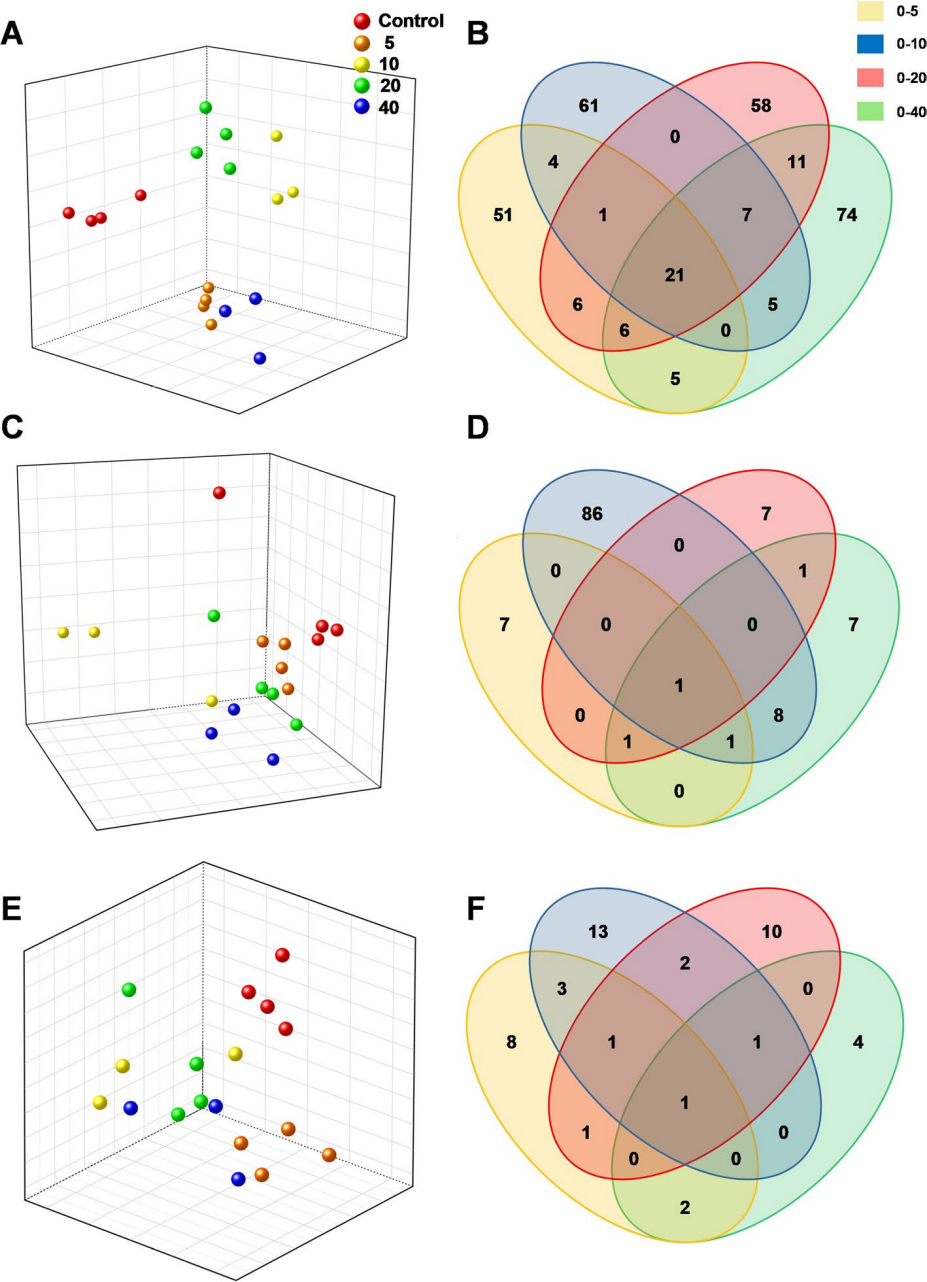
Differentially expressed transcripts (DETs) across treatments. nMDS plots display DETs of coral (**A**), Symbiodiniaceae (**C**), and prokaryotic microbes (**E**) based on the square root transformed log base 2 of counts per million reads via Bray-Curtis measurement of dissimilarity. Each symbol represents a replicate. Kruskal’s stress values are 0.04, 0.03 and 0.11 for A, C, E, respectively. Venn diagrams show the comparison of DETs of coral (**B**), Symbiodiniaceae (**D**), and prokaryotic microbes (**F**) in different treatments compared with the control

Different numbers of DETs were detected in coral meta-organism, including coral, Symbiodiniaceae, and prokaryotic microbes, respectively (Table 1). The overall differences in the comparisons amongst different treatments and the control for coral, Symbiodiniaceae, and prokaryotic microbes were illustrated by MA (ratio density) plots (Supplementary Figure S1, Additional File 1). The Symbiodiniaceae communities (D1 and D1.3) and taxonomic classification of prokaryotic transcripts (e.g., Janthinobacterium, Pseudomonas, Acinetobacter, and Stenotrophomonas) showed similar structures in each treatment and the control (Supplementary Table S3 and Figure S2, Additional File 1). The main prokaryotic genera detected all maintained features of nitrogen metabolism, including Janthinobacterium, Pseudomonas, and Acinetobacter, thereby inferring a functional role of the prokaryotic microbiome in the acclimation of the coral meta-organism to nitrate enrichment. An individual transcript that was differentially expressed in at least one treatment within pairwise differential expression analysis was regarded as a DET. Collectively, a total of 310 coral transcripts, 119 Symbiodiniaceae transcripts, and 46 prokaryotic transcripts were retained as DETs for further analysis. The Venn diagram illustrates the sharing of DETs in coral, Symbiodiniaceae, and prokaryotic microbes under nitrate enrichment (Fig. 1B, D, and F, respectively). Twenty-one coral, one Symbiodiniaceae, and one prokaryotic DETs were shared across all treatment comparisons. The non-parametric multidimensional scaling (nMDS) indicates that the DETs of corals in different samples were well separated by nitrate concentration (Fig. 1A). There was no significant difference amongst samples from the same group. However, DETs of Symbiodiniaceae and prokaryotes did not show a clear gradient through varying nitrate concentrations (Fig. 1C and E).

**Table 1:** Numbers of differentially expressed transcripts in coral meta-organism under different treatments

		Control-5	Control-10	Control-20	Control-40
Coral	Upregulated	41	49	57	63
	Downregulated	53	50	53	66
Symbiodiniaceae	Upregulated	4	8	3	9
	Downregulated	6	88	7	10
Prokaryotic microbes	Upregulated	3	9	9	1
	Downregulated	13	12	7	7

### Function partitioning and diversity of coral, Symbiodiniaceae, and prokaryotic microbes under different nitrate treatments

In coral, the shared transcripts were mainly related to the development, including cell differentiation, cell growth, and anatomical structure development; stress response; and protein metabolism (Figure S3A, Additional File 1), thereby suggesting that coral larvae development was affected by nutrient stress. Meanwhile in Symbiodiniaceae, the shared transcripts largely belonged to DNA/RNA metabolism, transport of protein and ions, stress response, and development (Figure S3B, Additional File 1). In prokaryotic microbes, transcripts for DNA metabolism and the transport of lipid and ion comprised most of the shared transcripts (Figure S3C, Additional File 1). When mapping the DETs to pathways in KEGG and Reactome databases, large numbers of coral DETs were assigned to SMAD2/3:SMAD4 transcriptional activity related to the development and HSF1 regulation related to stress response (Table S4, Additional File 1). Nitrogen metabolism was also found to be related to coral DETs. In addition, Symbiodiniaceae had DETs mainly related to development, including pathways of brassinosteroid signalling and transport and synthesis of PAPS, as well as symbiotic relationship, such as pathways of neutrophil degranulation and strigolactone signaling. Nitrate and ammonia assimilation pathways were also detected among Symbiodiniaceae DETs (Table S5, Additional File 1). The DETs of prokaryotic microbes were related to carbon fixation, oxidative phosphorylation, and amino/nucleotide sugar metabolism (Table S6, Additional File 1).

Gene set enrichment analysis (GSEA) considered more transcripts and thus could form a more complete picture of the functional differences. Based on the GSEA of corals, 9, 26, 22, and 15 transcript sets were upregulated and 1, 20, 41, and 0 transcript sets were downregulated in the 5, 10, 20, and 40 µM groups, respectively (FDR ≤ 0.05). For Symbiodiniaceae transcripts, 48, 100, 50, and 70 transcript sets were upregulated and 1, 62, 8, and 50 transcript sets were downregulated in the 5, 10, 20, and 40 µM groups, respectively (FDR ≤ 0.05). Meanwhile for prokaryotic transcripts, 2, 15, 2, and 10 transcript sets were upregulated and 2, 0, 2, and 1 transcript sets were downregulated in the 5, 10, 20, and 40 µM groups, respectively (*p* ≤ 0.001).

**Fig. 2:**
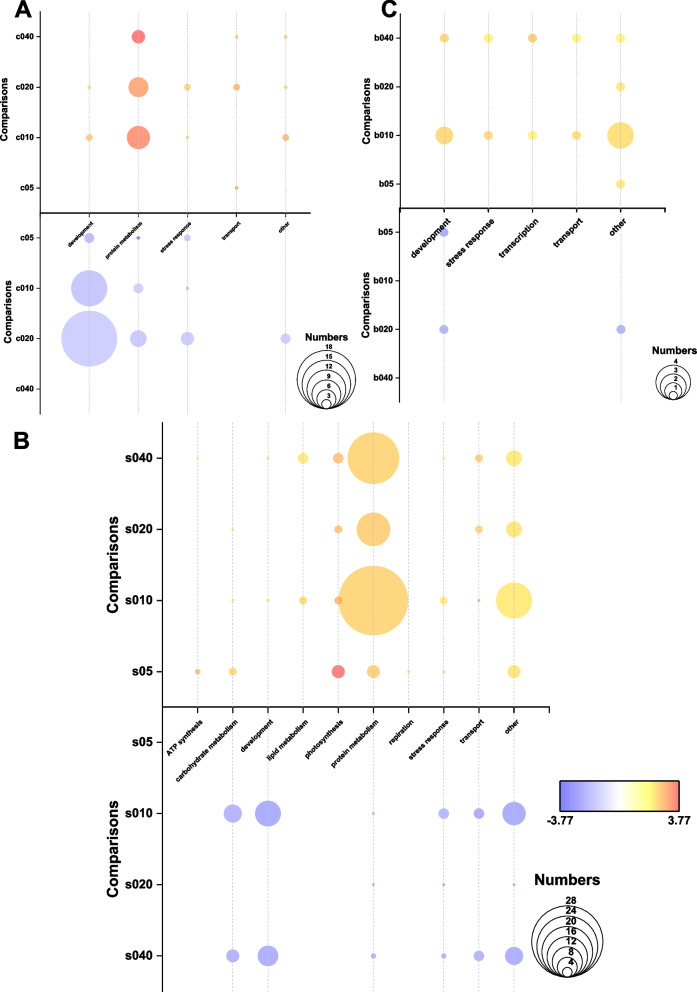
GSEA results of coral meta-organism transcriptome under different treatment conditions. GSEA present the major enriched transcript sets of coral (**A**), Symbiodiniaceae (**B**), and prokaryotic microbes (**C**) transcriptome at varying nitrate concentrations. The node size represents numbers of enriched transcript sets in certain functional category. The color represents the averaged NES values of enriched transcript sets

The biological processes of enriched transcript sets in coral showed that coral larvae development, including pigmentation, cell development, and differentiation, was downregulated in all treatments except 40 µM (Fig. 2A, Table S7, Additional File 1). In particular, the 20 µM group exhibited the largest numbers of downregulated transcript sets related to development, which mostly involves cell division and differentiation, with lowest NES values, whilst coral transport of xenobiotics was shown to be more upregulated than that in other treatments. The transport-related transcript sets in Symbiodiniaceae were less upregulated than those in the coral. Moreover, Symbiodiniaceae development (mainly comprising cell division and growth) and carbohydrate metabolism were downregulated in the 10 and 40 µM groups (Fig. 2B, Table S8, Additional File 1). In contrast to Symbiodiniaceae development, prokaryotic microbe development, including growth and cell division, was upregulated in the 10 and 40 µM groups but decreased in the two other treatments (Fig. 2C, Table S9, Additional File 1). Finally, coral and Symbiodiniaceae enriched different sets of stress response transcripts in all treatments, mainly including immune and stimulus responses (Table S7, S8, Additional File 1).

#### Associations amongst coral meta-organisms under different nitrate enrichments

**Fig. 3:**
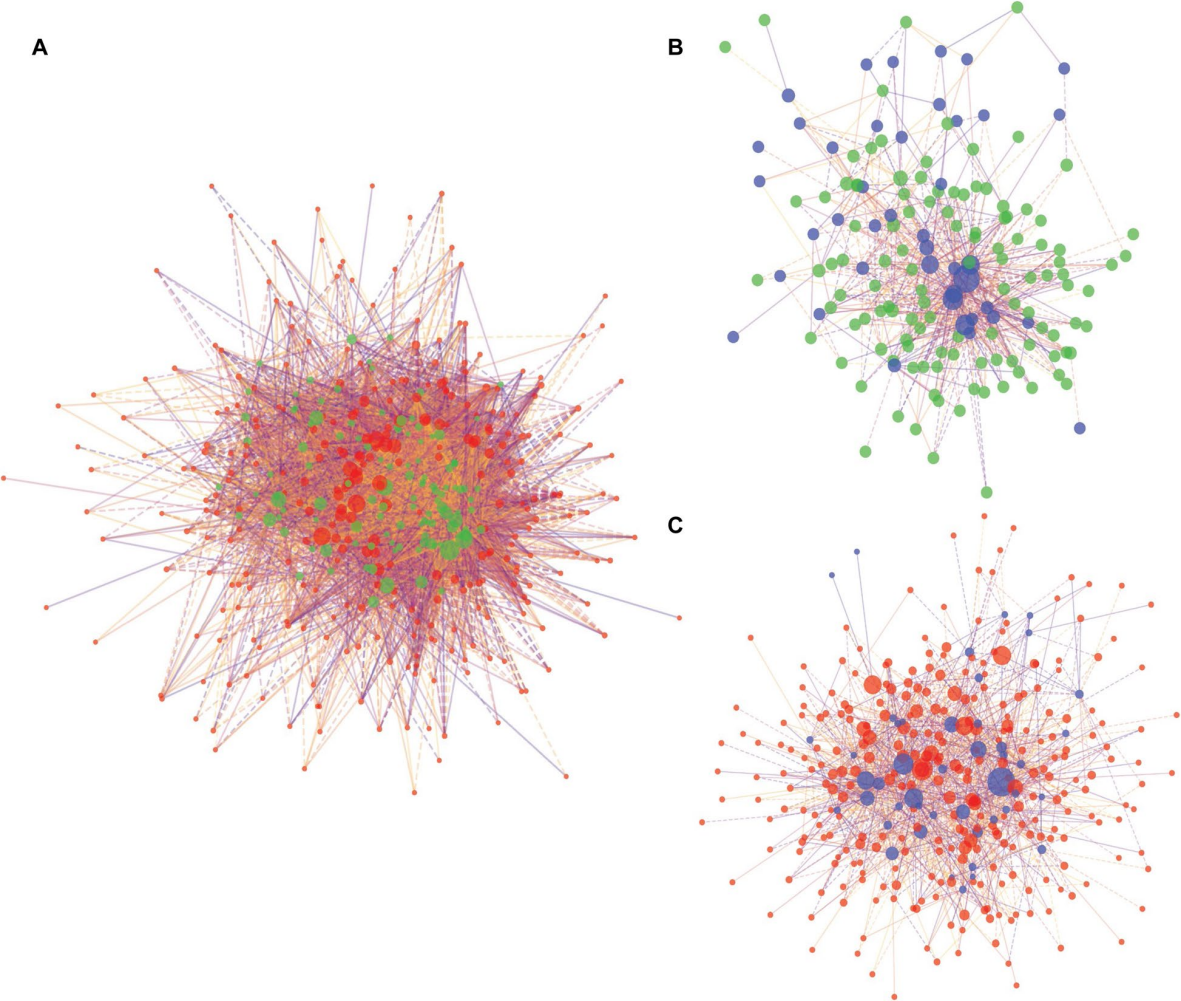
Correlations amongst differentially expressed transcripts (DETs) in coral meta-organism under nitrate enrichments. Organic correlation network visualizing significant correlations between transcripts of coral and Symbiodiniaceae (**A**), Symbiodiniaceae, and prokaryotic microbes and (**B**) those of coral and prokaryotic microbes (**C**). Red, green, and blue represent coral, Symbiodiniaceae and prokaryotic transcripts, respectively. Node size reflects the adjusted betweenness centrality of the variable. Line types (solid = positive and dashed = negative) indicate the Spearman correlation coefficient

The co-expression network of DETs in coral meta-organisms showed compact, organised networks representing coral-algal, algal-prokaryotic, and coral-prokaryotic associations (Fig. 3). In total, 7,658 out of 36,890, 627 out of 5,474, and 1,373 out of 14,260 significant correlations (*p* ≤ 0.05) were detected in the coral-algal, algal-prokaryotic, and coral-prokaryotic networks, respectively. The highest proportion of significant correlations was found in the coral-algal network (20.76%); meanwhile, the proportion of significant correlations in the algal-prokaryotic network (11.45%) was slightly higher than that in the coral-prokaryotic network (9.63%). In the coral-algal network, coral, and Symbiodiniaceae maintained similar values of the largest betweenness centralities of the transcripts. In the algal-prokaryotic and coral-prokaryotic networks, the prokaryotic transcripts had the largest betweenness centralities amongst the transcripts of the top values, particularly in the algal-prokaryotic network. The top 20 transcripts with the highest betweenness centrality in each network were regarded as core transcripts. In the coral-algal network, the core transcripts of coral mainly belonged to development and transport, whereas the core transcripts of Symbiodiniaceae were mostly related to transport and nitrogen compound metabolism (Figures S4A and B, Additional File 1). In the algal-prokaryotic network, the Symbiodiniaceae core transcripts were principally associated with defence response, substance metabolism, and symbiotic interaction. In addition, the core transcripts belonging to prokaryotic microbes were associated with transport and nitrogen compound metabolism (Figures S4C and D, Additional File 1). In the coral-prokaryotic network, the coral core transcripts largely maintained substance metabolism and stress response functions; meanwhile, the core transcripts of prokaryotic microbes were mainly related to transport and nitrogen compound metabolism (Figures S4E and F, Additional File 1).

**Fig. 4:**
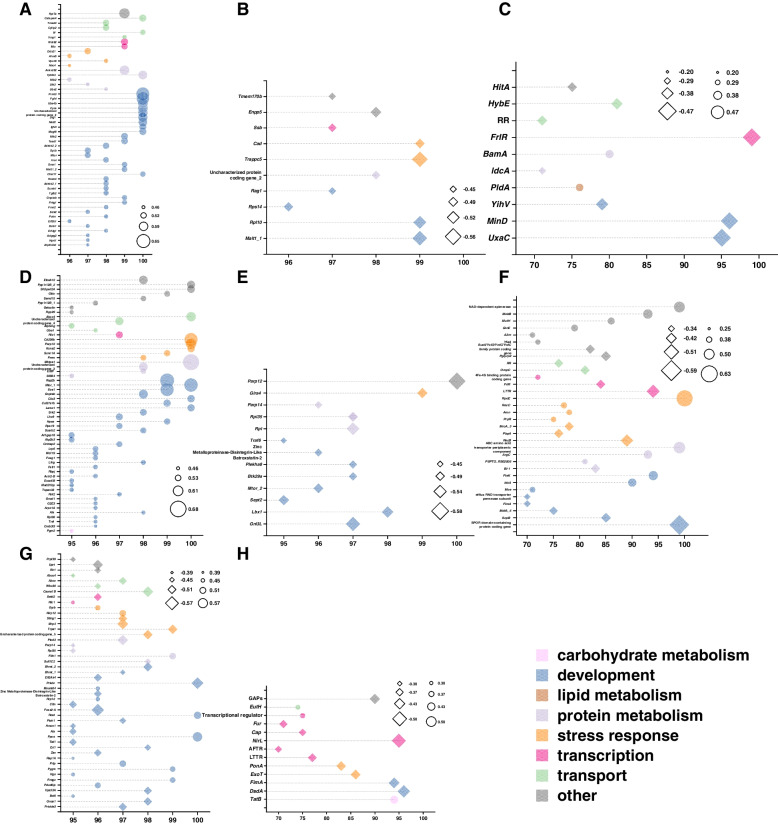
GLMMLasso fitting between coral/prokaryotic transcripts and Symbiodiniaceae physiology. GLMMLasso fitting indicates correlations between the enriched coral transcript and Symbiodiniaceae density (**A**, **B**), photosynthesis (**D**, **E**), and respiration (**G**), similar to enriched prokaryotic transcripts (**C**, **F**, and **H**). Herein, the symbol size demonstrates average estimates from total runs of identified significant correlated transcripts. The circle and square represent positive and negative correlations, respectively. Different colors represent different functional categories. *X* and *Y* axes of each panel represent transcripts and run numbers, respectively

The average Symbiodiniaceae density in *P*. *damicornis* larvae, ranging from 1.6 × 10^4^ to 1.9 × 10^4^ cells per larvae, increased in all treatments as compared with the control; however, the analysis of variance (ANOVA) showed it did not significantly differ amongst treatment groups (*p* = 0.05, Table 2, Figure S5A, Additional File 1). Herein, the *P*_G_ increased when larvae were exposed to nitrate enrichment in general and it was the highest in the 5 µM group, slightly increased in the 10 and 20 µM groups and moderately increased in the 40 µM group (Figure S5B, Additional File 1). In addition, the generalized linear mixed model using least absolute shrinkage and selection operator (the GLMMLasso model) further simulated the correlations between the physiological parameters (density, photosynthesis, and respiration) of Symbiodiniaceae and the coral/prokaryotic-enriched transcripts obtained from GSEA results (Fig. 4). The model showed that Symbiodiniaceae density, photosynthesis, and respiration were all correlated with changes in coral development transcripts including both positive and negative relationships; Symbiodiniaceae respiration had the largest number of negative correlations with coral development transcripts. Furthermore, Symbiodiniaceae respiration had the most negative correlations with coral transcripts assigned to transport functions. Meanwhile, unlike coral transcripts, prokaryotic transcripts mostly exhibited negative correlations with the physiological parameters of Symbiodiniaceae. There were three prokaryotic transcripts related to the development found to have positive correlations when Symbiodiniaceae photosynthesis was applied. The prokaryotic transcripts positively correlated with the physiological parameters of Symbiodiniaceae were mainly related to protein and lipid metabolism. Detailed information on significantly correlated transcripts detected in the GLMMLasso model is shown in Table S10 and S11, Additional File 1.

**Table 2:** Statistics results of ANOVA

Comparisons	*F* value	Prob > *F*
Control-5	0.05	0.82
Control-10	0.69	0.41
Control-20	1.53	0.23
Control-40	0.06	0.81

**Fig. 5:**
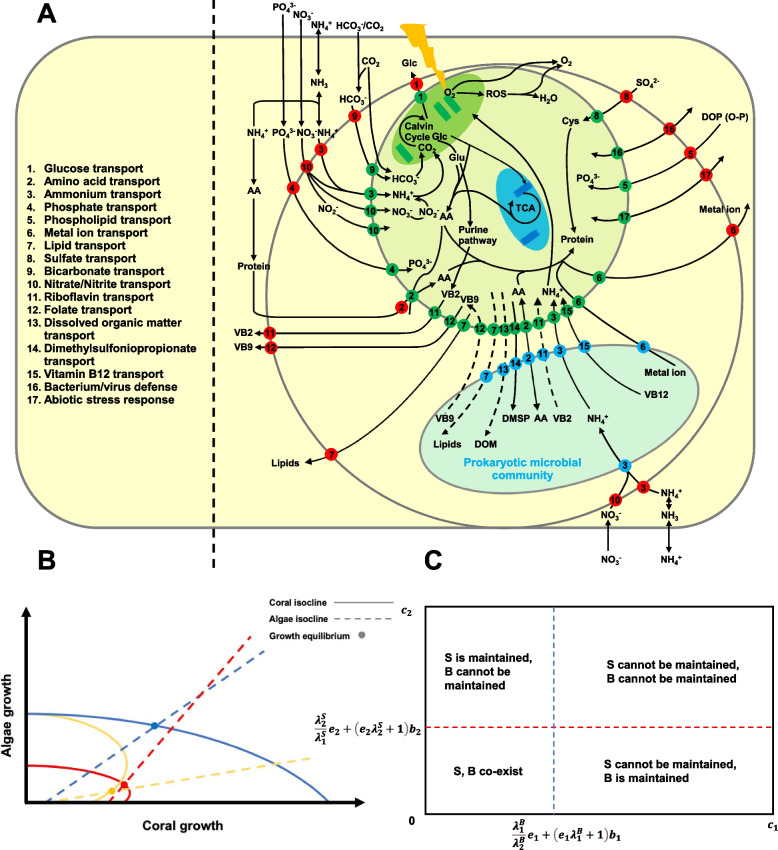
Coral meta-organism association-related pathways and analytical model for associations in coral meta-organism. Representing primary potential pathways related coral meta-organism associations (**A**, adapted from Lin et al. [40]), coral-algal association shifting (**B**), and algal-prokaryotic competition (**C**). Yellow, green, and blue parts represent coral host, Symbiodiniaceae community, and prokaryotic community, respectively. Small circles in red, green, and blue represent the transcripts potentially contributing to coral meta-organism associations in coral, Symbiodiniaceae, and prokaryotic microbes, respectively. ${\lambda }_{1}^{B}=\frac{1}{{e}_{1}}(\frac{{g}_{1}}{{d}_{1}}{\overline{H} }^{\overline{B} }-1)$, ${\lambda }_{2}^{B}=\frac{1}{{e}_{2}}(\frac{{g}_{2}}{{d}_{2}}{\overline{H} }^{\overline{B} }-1)$, ${\lambda }_{1}^{S}=\frac{1}{{e}_{1}}(\frac{{g}_{1}}{{d}_{1}}{\overline{H} }^{\overline{S} }-1)$, ${\lambda }_{2}^{S}=\frac{1}{{e}_{2}}(\frac{{g}_{2}}{{d}_{2}}{\overline{H} }^{\overline{S} }-1)$. ${\overline{H} }^{\overline{S} }$, ${\overline{H} }^{\overline{B} }$ represent coral biomass in boundary situations where the density of prokaryotic or Symbiodiniaceae community is zero, respectively. Other equation characters are the same as the analytical model characters explained in the discussion. Abbreviations: AA, amino acids; DOP, dissolved organic phosphorus; Glc, glucose; Glu, glutamate; ROS, reactive oxygen species; TCA, tricarboxylic acid cycle; VB2, vitamin B2 (riboflavin); VB9, vitamin B9 (folate); S, Symbiodiniaceae community; B, prokaryotic community

Based on previous genomic research on coral, Symbiodiniaceae, and prokaryotic microbes [40–44], the present study identified 17 kinds of transcript sets that potentially maintain associations in coral meta-organisms. This includes material transport and stress response, which were identified from the total coral meta-organism transcriptome. These transcript sets were used to propose a conceptual model to outline the nutrient pathways related to coral meta-organism associations under nitrate enrichments (Fig. 5A). Herein, several transcripts potentially contributing to algal-prokaryotic associations were also detected, including the transport of metal ions, amino acids, nitrogen compounds, Dimethysulfoniopropionate (DMSP), and vitamin B_12_ (Fig. 5A) [42–44].

## Discussion

### Transcriptomic response of coral under nitrate enrichments

Previous studies revealed that eutrophication could cause depressed coral reproduction and reduce calcification as well as skeleton density [45, 46]. However, several studies argued that nutrient enrichment does not directly affect coral physiology and that increased nutrients can promote coral growth in certain circumstances [47–49]. Nitrogen identity, nutrient ecological source, N to P ratio, and coral specie characteristics all affect coral performance under eutrophication [7, 9, 13]. In the present study, nitrate treatment did not affect larval survival, whilst elevated nitrate concentration induced several changes in protein metabolism and altered the stress responses of coral larvae. Coral transcriptome completeness was similar to that of previous research, but the completeness of the Symbiodiniaceae transcriptome was not [50, 51]. It is because whole coral larvae, with their associated microbiome, were used for RNA extraction. More complete transcripts are limited to Symbiodiniaceae in pure culture rather than the transcripts of an in hospite Symbiodiniaceae community analysis. Herein, the number of transcripts in the present study was much greater than that of the predicted genes and transcripts in a previous study [34] because of the potential for single genes to generate different transcripts. In addition, highly similar transcripts were retained in the present study to avoid information loss. Overall, larval development transcripts decreased in all treatments but the decrease in the 40 µM group was not significant. Given this response in the short-term larval stage explored in this study, developmental inhibition under long-term elevated nitrate concentrations may affect the resilience of coral reefs.

### Coral-algal associations altered under nitrate enrichments

Coral-algal and coral-prokaryotic associations are fundamental in extending the capacity of the coral as a meta-organism to face various environmental stressors [4]. The current study also found that nitrate enrichment affected the energy metabolism of the coral meta-organisms and the associated groups could establish a response mechanism to environmental stress. The nutrient exchange transcripts of coral, Symbiodiniaceae, and prokaryotic microbes were closely correlated and associated with coral development, thereby indicating that nutrient exchange has a critical role in coral host acclimation to nitrate enrichments. Although the nitrogen metabolism-related pathway was detected to be related to coral DETs, there was no core coral transcript related to nitrogen metabolism. In contrast, Symbiodiniaceae and prokaryotic microbes had nitrogen metabolism among their core transcripts in the coral–algal and coral–prokaryotic networks. Consistent with the results of previous studies [14, 20, 52], metabolising nitrogen compounds of Symbiodiniaceae and prokaryotic microbes appears to be of central importance in coral-microbial relationships.

In the present study, correlation networks implied that the associations between coral and Symbiodiniaceae were much stronger than algal-prokaryotic and coral-prokaryotic associations. Previous studies revealed that coral control Symbiodiniaceae in three manners: (1) a set of free amino acids in coral tissue stimulates Symbiodiniaceae to release their photosynthates [53, 54], (2) coral digests/degrades Symbiodiniaceae to control Symbiodiniaceae density [55], and (3) coral limits the nutrient uptake of Symbiodiniaceae [31, 56]. Herein, in the 5 µM group, the average Symbiodiniaceae density slightly increased but *P*_G_ and *P*_N_ were the highest, thereby suggesting that the photosynthetic efficiency of Symbiodiniaceae in the 5 µM group was substantially enhanced when nitrate concentration was slightly elevated. However, further increases in nitrate concentration led to Symbiodiniaceae overgrowth without increased photosynthetic efficiency. In addition to photophysiology, transcriptome results showed the 5 µM group enhanced most ATP synthesis, carbohydrate metabolism, and photosynthesis. These findings indicated that the coral larvae may have regulated its Symbiodiniaceae density by digesting the algae or limiting the nitrogen availability to avoid Symbiodiniaceae overgrowth and to enhance the photosynthetic rate to meet the high energy requirement under nitrate enrichment. The 5 µM group showed less enriched coral larval transcripts for protein regulation and larval development than the 20 µM group. At a nitrate concentration of 10 µM, the Symbiodiniaceae density increased; however, the photosynthetic rate decreased compared with that at 5 µM. The Symbiodiniaceae density in the 20 µM group was the highest amongst all treatments, whilst the photosynthetic rate was lower than that in the 5 µM group. Various coral protein regulation transcripts, mostly involved in protein modification such as phosphorylation and trimethylation, and most stressor response transcripts were enriched in the 20 µM group, thus suggesting that the coral larvae were substantially impaired under this concentration. Symbiodiniaceae tend to transport less subsidy to the coral host and cheat the host with nutrients when under stress [57, 58]. In this study, coral larval development was largely downregulated in the 20 µM group, thus indicating a similar shift in the coral-algal associations from mutualism towards parasitism [59, 60]. When the nitrate concentration was increased to 40 µM, the Symbiodiniaceae density was lower than that of the 20 µM group. Despite the high nitrate concentration, coral larvae development and stress response transcripts were not significantly enriched, thereby suggesting coral-algal associations were not harmfully affected in the 40 µM group and restored compared to the 20 µM group. Assigning Symbiodiniaceae DETs to pathways further revealed alterations to the coral-algal symbiotic relationship under different nutrient treatments.

In addition to mapping the functional roles of members of the coral meta-organism, we developed an analytical model based on the Lotka-Volterra model and symbiosis model [61]. The application of this model demonstrates how coral associations can be altered under nitrate enrichments (Eqs. 1- 3).

1 $$\frac{dH}{dt}=rH\left(1-\frac{H}{K+{\gamma }_{1}S+{\gamma }_{2}SB}\right)-{a}_{1}SH-{a}_{2}BH$$

2 $$\frac{dS}{dt}={g}_{1}HS\left(1+{b}_{1}B\right)-{d}_{1}S\left(1+{e}_{1}S+{c}_{1}B\right)$$

3 $$\frac{dB}{dt}={g}_{2}HB\left(1+{b}_{2}S\right)-{d}_{2}B\left(1+{e}_{2}B+{c}_{2}S\right)$$

In these equations, H represents the biomass of coral; S and B represent densities of Symbiodiniaceae community and prokaryotic community, respectively; $r$ represents coral independent growth rate; K represents environmental capacity; ${\gamma }_{i}$ represents the benefits that Symbiodiniaceae/prokaryotic microbes provide to the coral host; ${a}_{i}$ represents the cost of Symbiodiniaceae/prokaryotic microbes from the coral host; ${g}_{i}$ represents the independent growth rate of Symbiodiniaceae and prokaryotic microbes; ${b}_{i}$ represents the benefits that Symbiodiniaceae/prokaryotic microbes receive from each other; ${d}_{i}$ and ${e}_{i}$ represent the independent/dependent death rate of Symbiodiniaceae/prokaryotic microbes, respectively; and ${c}_{i}$ represents the competition between Symbiodiniaceae and prokaryotic microbes.

Our analytical model underpins the growth equilibriums of coral-algal associations presented in Fig. 5B. The intersection of the coral and algae isocline (growth equilibrium) defines the limit under which the coral-algal association is in mutualism; otherwise, the association is expected to shift to parasitism. Under nitrate enrichment, Symbiodiniaceae can retain more nutrients for cell division and growth pushing the association above this intersection wherein symbionts retain nutrients for their own replication, decrease their contributions to the mutualism, and ultimately can become parasitic. This is consistent with previous studies wherein environmental stress, including thermal stress and nutrient enrichment can drive unstable symbiosis or parasitism in coral-algal associations [7, 13, 57, 58, 62].

### Algal-prokaryotic associations participate in coral meta-organism acclimation under nitrate enrichments

Free-living Symbiodiniaceae live in association with prokaryotic microbes [63, 64], thus suggesting that prokaryotic microbes can provide essential metabolic resources for the survival of Symbiodiniaceae in the oligotrophic environment. In this role, prokaryotic microbes have been suggested to regulate the functional diversity, competitive performance, and nutrient acquisition of Symbiodiniaceae [63, 65, 66]. Beneficial associations between Symbiodiniaceae and prokaryotic microbes were also detected in the present study. For example, genomic evidence showed that Symbiodiniaceae acquire vitamin B_12_, which is an essential cofactor for algal growth, from associated prokaryotic microbes [42].

Moreover, Symbiodiniaceae and prokaryotic microbes are critical for maintaining and building up the resistance of coral meta-organism health [14, 18, 33, 67] and that algal-prokaryotic associations could be necessary to nutrient cycling and competitive fitness of coral meta-organisms [68]. In the present study, associations amongst coral meta-organism were affected by eutrophication; meanwhile, correlations between Symbiodiniaceae and prokaryotic microbes could contribute to coral larval acclimation. Prokaryotic microbes take up DMSP released by Symbiodiniaceae [69] or can directly produce DMSP [70], which is crucial for antioxidant scavenging and pathogen defence via the synthesis of antibiotics [71, 72]. Several prokaryotic microbes that generally co-occurred with Symbiodiniaceae maintain the key functions of nutrient metabolism; particularly, diazotrophs provide Symbiodiniaceae with fixed nitrogen for cell division and growth [68, 69]. The present work consistently showed that the core transcripts of prokaryotic microbes involved in algal-prokaryotic interactions were related to transport and nitrogen compound metabolism, thereby suggesting an intimate nutrient exchange between Symbiodiniaceae and prokaryotic microbes continues to occur within coral meta-organism under nitrate enrichments.

Prokaryotic microbes are also capable of inhibiting algal growth via lysis and nutrient competition [73]. Several studies have found that prokaryotic microbes could inhibit algae by releasing extracellular compounds, such as biosurfactants and alkaloids [74, 75]. Limited studies have considered algal-prokaryotic competition within coral meta-organisms; though some demonstrated that pathogen overgrowth would occur under certain conditions that impaired Symbiodiniaceae and eventually led to coral bleaching [76]. In the present study, Symbiodiniaceae in 10 and 40 µM groups showed downregulation of many development related transcript sets, mainly associated with cell development and division, with the upregulation of prokaryotic microbial growth transcript sets. Meanwhile, Symbiodiniaceae development was not affected with no related transcript set significantly enriched in 5 and 20 µM groups as the growth of prokaryotic microbes decreased. Similarly, the GLMMLasso results showed that several prokaryotic transcripts, including transcripts related to growth and transport, were negatively correlated with Symbiodiniaceae density and respiration. In particular, all growth-related prokaryotic transcripts were negatively correlated with Symbiodiniaceae density. These findings could result from competitive relationships between Symbiodiniaceae and prokaryotic microbes for nitrate. Ultimately, the coral larval development was more downregulated in the 5 and 20 µM groups than in other groups but was restored in the 40 µM group, thus inferring that the competition between prokaryotic microbes and Symbiodiniaceae may control Symbiodiniaceae and benefit coral larval development under high nitrate concentrations. Therefore, a balanced coexistence of Symbiodiniaceae and prokaryotic microbes is likely optimal for coral larval acclimation under nitrate enrichments (Fig. 5C). Our analytical model predicts that the competition between Symbiodiniaceae and prokaryotic microbes should occur within certain ranges for Symbiodiniaceae and prokaryotic microbes to coexist (Fig. 5C), which could ensure proper nutrient exchange between the two partners and stable community densities. Within this model, coral-algal associations will be mutualistic when growth rates of Symbiodiniaceae and prokaryotic microbes are maintained at proper rates with a low equilibrium density of prokaryotic microbes (Fig. 5B, Table S12, Additional File 1).

## Conclusion

*P. damicornis* larvae, Symbiodiniaceae, and prokaryotic microbes displayed various transcriptomic responses to nitrate enrichments. Under nitrate enrichments, transcript sets relevant to the development of coral, Symbiodiniaceae and prokaryotes were altered. GLMMLasso model fitting detected significant correlations between Symbiodiniaceae physiology and coral/prokaryotic transcripts. These patterns are in line with a change in the coral-algal associations, more specifically a shift from mutualism towards parasitism wherein Symbiodiniaceae retained more nutrients for self-growth under high nitrate concentrations, leading to impaired coral larval development. Meanwhile, prokaryotic microbes might mediate this interaction by competitively restraining Symbiodiniaceae growth and allowing for partial restoration of coral larval development. Whilst this study focused on the early life stages of a coral, the results highlight the potential significance of algal-prokaryotic associations in coral meta-organisms under eutrophic conditions. Future studies shall explore more characteristics of prokaryotic microbes, particularly algal-prokaryotic associations, and more directly assess the dynamics of Symbiodiniaceae and prokaryote competition, which will fill the gap on the functional role of prokaryotic microbes in coral larval health and settlement and improve our understanding of the response of coral meta-organisms to eutrophication more generally. Overall, the present study provides a knowledge base for coral reef conservation and suggested that nutrient enrichment management could have a remarkable effect on coral recruitment.

## Methods

### Study area and sample collection

Luhuitou fringing reef is located in southeast Sanya Bay of Hainan Island in the South China Sea (Supplementary Figure S6, Additional File 1). The reef is approximately 3 km long and 250-500 m wide. It receives sewage discharge and runoff from Sanya Bay and Sanya River; thus, it is largely influenced by anthropogenic activities. The nutrient level in the Luhuitou fringing reef is enriched, the concentrations of nitrate ranging from 1.68 μmol L^−1^ to 5.74 μmol L^−1^ in the wet seasons and dry seasons [77], which is much higher than that in the typical oligotrophic waters of Indo-Pacific coral reefs with only 0.03 μmol L^−1^ [78]. The live coral coverage on the Luhuitou fringing reef has extensively declined owing to anthropogenic effects and global climate change from 85% in the 1960s to 10% in 2014 [2, 79].

A total of ten apparently healthy colonies of adult *P. damicornis* were randomly collected at a depth of ∼2 m from the Luhuitou fringing reef and immediately transferred to the nearby CAS-HKUST Sanya Joint Laboratory of Marine Science Research. All colonies were individually placed in larval collection apparatuses, with running sand-filtered seawater pumped from ∼3 m depth in front of the lab. Subsequently, planula larvae from different colonies were released before the new moon. Then, they were pooled and randomly assigned to each of the five experimental treatments.

### Experimental setup

The nitrate enrichment experiment consisted of five treatments. The control aquaria were supplied with 0.5 µm filtered natural seawater, whilst the four nutrient-enriched treatment aquaria were achieved through mixing with potassium nitrate at target concentrations of 5, 10, 20, and 40 µM. The control nitrate concentration was 2.5 µM, reflecting the fringing reef environment from which the corals were collected. Each treatment contained four replicates of 350 mL aquaria, equipped with a recirculating pump. To measure the seawater nitrate level in each aquarium, 30 mL seawater samples were taken daily from the aquaria, filtered through GFF filters, and frozen before analysis. The seawater samples from the experiment and the collection site were measured using a nutrient autoanalyzer (Seal Analytical AA3, Germany). The actual nitrate concentrations during the experiment are given in Table S13 in Additional File 1. The aquaria were illuminated with T5 fluorescent bulbs (Giesemann, Germany) at a mean irradiance of 300 µmol photons m^2^/s during a 12:12-h light: dark cycle, which is similar to the in situ light conditions from ∼2 m depth on the Luhuitou fringing reef, whereby the seawater temperature of the system was kept constant at 29 °C by using temperature controllers (WEIPRO, China). The experiment was kept under control conditions for 5 days and ended just before larval settlement.

A total of 20 larvae were randomly sampled for physiological measurements from each aquarium, as described below at the end of the experiment. The remaining larvae were aliquoted (approximately 20 larvae per aliquot) and preserved at − 80 °C for transcriptomic analyses.

### Photosynthesis, respiration, and symbiont density

There was a total of 20 larvae from each aquarium that were randomly chosen and transferred to a 2 mL glass chamber containing seawater from the respective incubation aquarium. The rates of net photosynthesis (*P*_N_) and dark respiration (*R*_D_) of larvae in each treatment were measured in the chambers. The chambers were equipped with magnetic stir bars and a temperature-compensated oxygen mini-sensor (Ocean Optics, USA) connected to a four-channel microsensor oxygen metre (PreSens, Germany). In addition, the chambers with seawater were used as blank controls for each treatment. *P*_N_ was measured at midday whereas *R*_D_ was measured in darkness after 2 h of dark adaptation in the chambers. Both the *P*_N_ and *R*_D_ were calculated by regressing oxygen production/consumption against 10-min measurement time and expressed as nmol O_2_ larvae^−1^ min^−1^. The gross photosynthesis (*P*_G_) was obtained by adding *R*_D_ and *P*_N_. Herein, after the photosynthetic and respiratory measurements were completed, the larvae were preserved at − 80 °C for symbiont density measurements.

Algal symbiont density was determined following the method described by Wiedenmann et al. [29]. Briefly, the larvae were homogenised using a pestle and resuspended in 200 µL autoclaved filtered seawater. Each 40 µL homogenate was loaded onto a haemocytometer to quantify the cell number. The algal symbiont density was calculated as the number per larva.

### Transcriptome sequencing

The total RNA was extracted from aliquots of 20 *P. damicornis* larvae from each replicate by using TRIzol (Invitrogen) following the manufacturer’s instructions. RNA quantity and integrity were assessed by 1% gel electrophoresis and an Agilent 2100 bioanalyzer (Agilent Technologies, USA), respectively. cDNA libraries were prepared from 3 μg of RNA per sample by using NEBNext UltraTM RNA Library Prep Kit for Illumina (NEB, USA) also following the manufacturer’s recommendations. The pooled and barcoded libraries were sequenced on the HiSeq PE150 Illumina sequencing platform by Novogene Bioinformatics Technology Co., Ltd., Beijing, China (www.novogene.cn).

### Transcriptome assembly

After low-quality reads were removed, the data from all samples were used to generate a total coral meta-organism reference transcriptome with Trinity 2.8.5 [80]. The coral meta-organism transcriptome was blasted using BLASTn against the Symbiodiniaceae ITS2 database [81], with *E*-values of 1e^−50^, to identify the Symbiodiniaceae types in the coral larvae and generate ITS2 gene references. Then, *P. damicornis* and Symbiodiniaceae transcriptomes were created by blasting the meta-organism transcriptome via BLASTx against the NCBI scleractinia and Symbiodiniaceae databases, respectively, with an *E*-value of 1e^−50^ and a 33 bp amino acid cutoff length. Furthermore, the resulting scleractinia reference transcriptome was blasted against the Symbiodiniaceae database with the same parameters and blasted reads were removed to generate the final *P. damicornis* transcriptome, thereby ensuring all transcripts were from *P. damicornis*. The final *P. damicornis* transcriptome completeness was assessed using BUSCO [82]. The Symbiodiniaceae transcriptome was similarly treated to remove ambiguous transcripts and establish a pure Symbiodiniaceae transcriptome. The prokaryotic transcriptome was firstly identified by Kraken 2 and then using BLASTx against NCBI bacteria and archaea database with an *E*-value of 1e^−5^, thus constituting the final prokaryotic transcriptome. Prokaryotic community in each sample was structured by Kraken2 with transcriptome data. GO terms were retrieved by performing InterPro analysis, EggNOG mapper, and GO mapping and annotation via Blast2GO under default parameters [83]. Enzyme codes were identified by mapping GO terms to EC-codes using Gene Ontology Consortium. Related pathways were retrieved by linking protein, GO IDs as well as enzyme codes to KEGG and Reactome databases [84–86].

### Statistical analysis

Each sample was mapped and counts against ITS2, *P. damicornis*, Symbiodiniaceae, and prokaryotic final transcriptomes were calculated by RSEM (RNA-Seq by Expectation-Maximization) to estimate the Symbiodiniaceae community and functional response of coral meta-organisms to nutrient stress [87]. Pairwise differential expression analysis of each treatment against the control was established using edgeR, with a logFC of more than 1 or less than − 1 and *p* values of 1.0 *E*-4 and 1.0 *E*-3 to identify differentially expressed coral/Symbiodiniaceae and prokaryotic transcripts, respectively. MA plots were created to visualize pairwise analysis for coral, Symbiodiniaceae, and prokaryotic microbes. The counts per million reads for each transcript differentially expressed in at least one pairwise analysis were used to perform nMDS in Primer-e and generate Venn diagrams. Gene set enrichment analysis was performed for each pairwise differential expression to measure the coral, Symbiodiniaceae, and prokaryotic microbial functional alterations in each treatment compared with the control [88]. The numbers and averaged normalized enriched values for each main category of enriched functions in coral, Symbiodiniaceae, and prokaryotic microbes were used to establish bubble plots in OriginLab 2021 (OriginLab, USA) by measuring the functional performance of the coral meta-organisms in the treatments and control. Variations in Symbiodiniaceae density, net photosynthesis, and gross photosynthesis were analyzed in OriginLab. In addition, the Spearman coefficient was used to estimate the correlations amongst the DETs in coral meta-organism. Co-expression networks were profiled with significant correlations amongst coral meta-organisms (*p* ≤ 0.05) and analyzed in Cytoscape to visualize the correlations. The betweenness centrality of the transcript was amended by the numbers of included transcripts in each network, thus avoiding the effects of different transcript quantities on the betweenness centrality calculation. The transcripts with top 20 highest betweenness centrality were used to create GO graphs for visualization of function annotations. The core-enriched transcripts in coral and prokaryotic microbes from GSEA analysis were fitted with the GLMMLasso model [89] containing the physiological data of Symbiodiniaceae (including density, photosynthesis, and respiration rate). For each physiological parameter of Symbiodiniaceae, the transcripts were randomly ranked and fitted into the model by using 10 transcripts in order each time. The above process was repeated for 100 times. The coral and prokaryotic transcripts with estimates greater than the averaged random effects in more than 95 and 70 runs, respectively, were retained as significant interaction transcripts for graph generation in OriginLab.

## Supplementary Information

**Additional file 1: Table S1.** Statistics of transcriptome de novo assembly. **Table S2.** Pair reads per sample mapping against different reference transcriptome. **Table S3. **Symbiodiniaceae community structure in different treatments. **Table S4.** Pathways related to coral differentially expressed transcripts in different treatments. **Table S5.** Pathways related to Symbiodiniaceae differentially expressed transcripts in different treatments. **Table S6.** Pathways related to prokaryotic differentially expressed transcripts in different treatments. **Table S7.** Functions of corals enriched transcript sets in different treatments. **Table S8.** Functions of Symbiodiniaceae enriched transcript sets in different treatments. **Table S9.** Functions of prokaryotic microbes enriched transcript sets in different treatments. **Table S10.** Detailed information of significantly correlated coral transcripts with Symbiodiniaceae physiology detected in GLMMLasso model. **Table S11.** Detailed information of significantly correlated prokaryotic transcripts with Symbiodiniaceae physiology detected in GLMMLasso model. **Table S12.** Parameters for analytical model example. **Table S13.** Nitrate concentration of each treatment during the 5-days experiment. **Figure S1.** MA plots of different comparisons (control-5, control-10, control-20, control-40) in coral (A, B, C, D), Symbiodiniaceae (E, F, G, H) and prokaryotic microbes (I, J, K, L). **Figure S2.** Prokaryotic community structures at phylum (A) and genus level (B) by Kraken2. Only genera with top 20 highest relative abundance were presented in the legend. **Figure S3.** Functions of shared transcripts in different comparisons. Representing coral (A), Symbiodiniaceae (B) and prokaryotic (C) shared transcripts, respectively. **Figure S4.** Functions of core transcripts in correlation networks. Representing coral (A) and Symbiodiniaceae (B) core transcripts in coral-algal network; Symbiodiniaceae (C) and prokaryotic microbial (D) core transcripts in algal-microbial network; coral (E) and prokaryotic microbial (F) core transcripts in coral-microbial network. **Figure S5.** Algal density (A) and photosynthetic rate (B) of larvae from different groups. Each dot in A represents a single larva. Each box in A and B represent the Q1-Q3 range in each group, and the whiskers in each box represent Q1 - 1.5SD and Q3 + 1.5SD of each group. Q1 and Q3 are the first and third quartile, and SD is the standard deviation. The line in each box represents the mean value for each group. *P*_N_ and *P*_G_ in B represent the net photosynthetic rate and gross photosynthetic rate, respectively. **Figure S6.** Location of sampling area. Star indicates the sampling and researching area, CAS-HKUST Sanya Joint Laboratory of Marine Science Research. HN, Hainan Island.

## Data Availability

All raw sequence data have been submitted to NCBI under SRA accession number PRJNA611041.
