# Supplementary material for: Meta-organism gene expression reveals that the impact of nitrate enrichment on coral larvae is mediated by their associated Symbiodiniaceae and prokaryotic assemblages
Source: Microbiome. 2023 Apr 26;11:89. doi: 10.1186/s40168-023-01495-0 (PMC10131396; doi:10.1186/s40168-023-01495-0)
Supplement: Supplementary file 2 — Additional file 1: Table S1. Statistics of transcriptome de novo assembly. Table S2. Pair reads per sample mapping against different reference transcriptome. Table S3. Symbiodiniaceae community structure in different treatments. Table S4. Pathways related to coral differentially expressed transcripts in different treatments. Table S5. Pathways related to Symbiodiniaceae differentially expressed transcripts in different treatments. Table S6. Pathways related to prokaryotic differentially expressed transcripts in different treatments. Table S7. Functions of corals enriched transcript sets in different treatments. Table S8. Functions of Symbiodiniaceae enriched transcript sets in different treatments. Table S9. Functions of prokaryotic microbes enriched transcript sets in different treatments. Table S10. Detailed information of significantly correlated coral transcripts with Symbiodiniaceae physiology detected in GLMMLasso model. Table S11. Detailed information of significantly correlated prokaryotic transcripts with Symbiodiniaceae physiology detected in GLMMLasso model. Table S12. Parameters for analytical model example. Table S13. Nitrate concentration of each treatment during the 5-days experiment. Figure S1. MA plots of different comparisons (control-5, control-10, control-20, control-40) in coral (A, B, C, D), Symbiodiniaceae (E, F, G, H) and prokaryotic microbes (I, J, K, L). Figure S2. Prokaryotic community structures at phylum (A) and genus level (B) by Kraken2. Only genera with top 20 highest relative abundance were presented in the legend. Figure S3. Functions of shared transcripts in different comparisons. Representing coral (A), Symbiodiniaceae (B) and prokaryotic (C) shared transcripts, respectively. Figure S4. Functions of core transcripts in correlation networks. Representing coral (A) and Symbiodiniaceae (B) core transcripts in coral-algal network; Symbiodiniaceae (C) and prokaryotic microbial (D) core transcripts in algal-microbial network; coral (E) [file 40168_2023_1495_MOESM1_ESM.zip › Supplementary/Additional File 1.docx]

**Algal–prokaryotic associations maintain coral larval development under nitrate enrichments**

### Haoya Tong^1,2^, Fang Zhang^3,4^, Jin Sun^5^, Shelby E. McIlroy^6,7^ Weipeng Zhang^8^, Yan Wang^2^, Hui Huang^3,4^, Guowei Zhou^3,4*^, Pei-Yuan Qian^1,2,4*^

^1^ Southern Marine Science and Engineering Guangdong Laboratory (Guangzhou), Nansha, Guangzhou, China

^2^ Department of Ocean Science and Hong Kong Branch of the Southern Marine Science and Engineering Guangdong Laboratory (Guangzhou), The Hong Kong University of Science and Technology, Hong Kong SAR, China

^3^ CAS Key Laboratory of Tropical Marine Bio-resources and Ecology, South China Sea Institute of Oceanology, Institute of South China Sea Ecology and Environmental Engineering, Chinese Academy of Sciences, Guangzhou, China

^4^ CAS-HKUST Sanya Joint Laboratory of Marine Science Research and Hainan Key Laboratory of Tropical Marine Biotechnology, Tropical Marine Biological Research Station in Hainan, Chinese Academy of Sciences, China

^5^ Institute of Evolution and Marine Biodiversity, Ocean University of China, Qingdao, China

^6^ The Swire Institute of Marine Science, The University of Hong Kong, Hong Kong SAR, China

^7^ School of Biological Sciences, The University of Hong Kong, Hong Kong, Hong Kong SAR, China

^8^ College of Marine Life Sciences, Ocean University of China, Qingdao, China

**^*^Co-corresponding authors:**

**Guowei Zhou**, PhD

E-mail: zhougw@scsio.ac.cn

**Pei-Yuan Qian**, PhD

E-mail: [boqianpy@ust.hk](mailto:boqianpy@ust.hk)

**Tables**

Table S1 Statistics of transcriptome de novo assembly

**Table S2** Pair reads per sample mapping against different reference transcriptome

**Table S3** Symbiodiniaceae community structure in different treatments

**Table S4** Pathways related to coral differentially expressed transcripts in different treatments

**Table S5** Pathways related to Symbiodiniaceae differentially expressed transcripts in different treatments

**Table S6** Pathways related to prokaryotic differentially expressed transcripts in different treatments

**Table S7** Functions of corals enriched transcript sets in different treatments

**Table S8** Functions of Symbiodiniaceae enriched transcript sets in different treatments

**Table S9** Functions of prokaryotic microbes enriched transcript sets in different treatments

**Table S10** Detailed information of significantly correlated coral transcripts with Symbiodiniaceae physiology detected in GLMMLasso model

**Table S11** Detailed information of significantly correlated prokaryotic transcripts with Symbiodiniaceae physiology detected in GLMMLasso model

**Table S12** Parameters for analytical model example

**Table S13** Nitrate concentration of each treatment during the 5-days experiment

**Figure Legends**

**Figure S1** MA plots of different comparisons (control-5, control-10, control-20, control-40) in coral (A, B, C, D), Symbiodiniaceae (E, F, G, H) and prokaryotic microbes (I, J, K, L).

**Figure S2** Prokaryotic community structures at phylum (A) and genus level (B) by Kraken2. Only genera with top 20 highest relative abundance were presented in the legend.

**Figure S3** Functions of shared transcripts in different comparisons. Representing coral (A), Symbiodiniaceae (B) and prokaryotic (C) shared transcripts, respectively.

**Figure S4** Functions of core transcripts in correlation networks. Representing coral (A) and Symbiodiniaceae (B) core transcripts in coral-algal network; Symbiodiniaceae (C) and prokaryotic microbial (D) core transcripts in algal-microbial network; coral (E) and prokaryotic microbial (F) core transcripts in coral-microbial network.

**Figure S5** Algal density (A) and photosynthetic rate (B) of larvae from different groups. Each dot in A represents a single larva. Each box in A and B represent the Q1-Q3 range in each group, and the whiskers in each box represent Q1 - 1.5SD and Q3 + 1.5SD of each group. Q1 and Q3 are the first and third quartile, and SD is the standard deviation. The line in each box represents the mean value for each group. P_N_ and P_G_ in B represent the net photosynthetic rate and gross photosynthetic rate, respectively.

**Figure S6** Location of sampling area. Star indicates the sampling and researching area, CAS-HKUST Sanya Joint Laboratory of Marine Science Research. HN, Hainan Island.
