# Supplementary figures and images for: Meta-organism gene expression reveals that the impact of nitrate enrichment on coral larvae is mediated by their associated Symbiodiniaceae and prokaryotic assemblages
Source: Microbiome. 2023 Apr 26;11:89. doi: 10.1186/s40168-023-01495-0 (PMC10131396; doi:10.1186/s40168-023-01495-0)

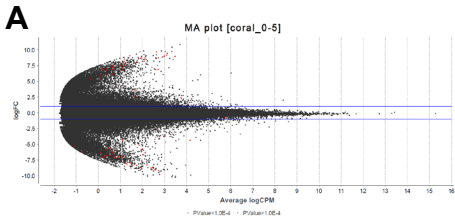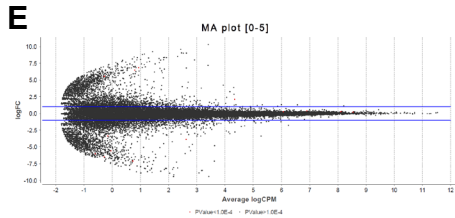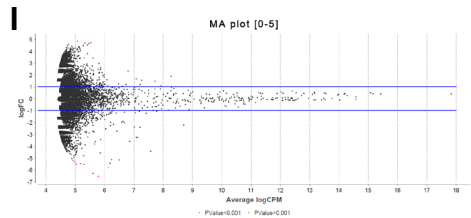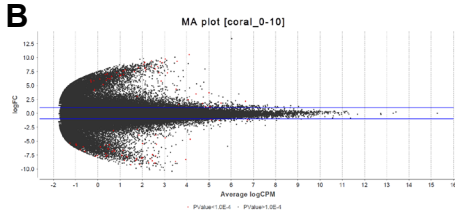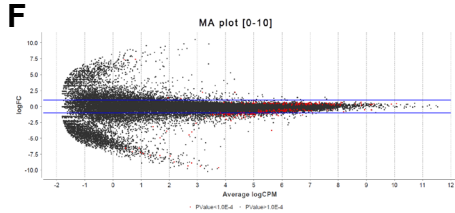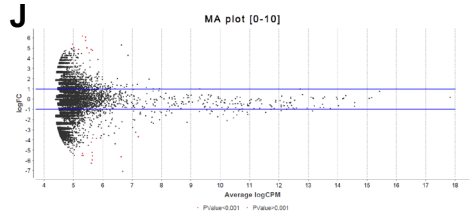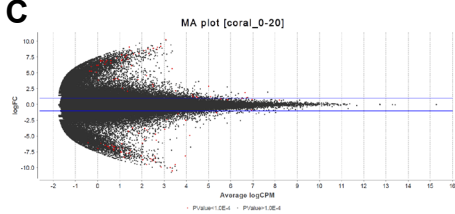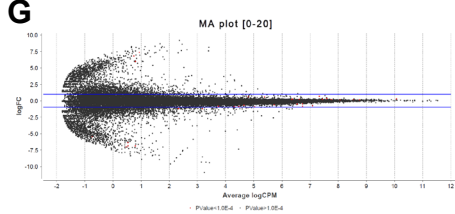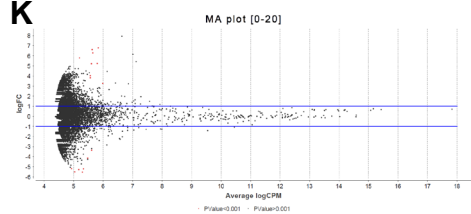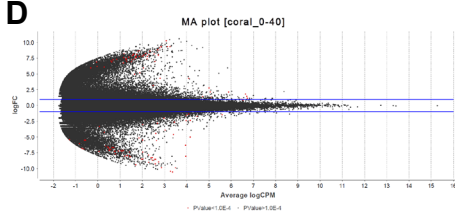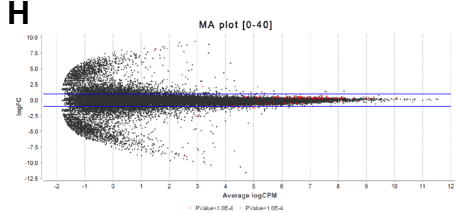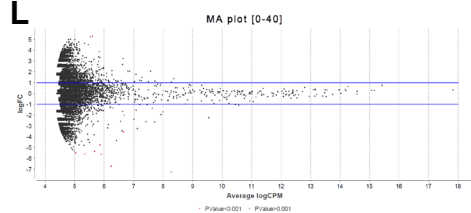

Supplement: Supplementary file 2 — Additional file 1: Table S1. Statistics of transcriptome de novo assembly. Table S2. Pair reads per sample mapping against different reference transcriptome. Table S3. Symbiodiniaceae community structure in different treatments. Table S4. Pathways related to coral differentially expressed transcripts in different treatments. Table S5. Pathways related to Symbiodiniaceae differentially expressed transcripts in different treatments. Table S6. Pathways related to prokaryotic differentially expressed transcripts in different treatments. Table S7. Functions of corals enriched transcript sets in different treatments. Table S8. Functions of Symbiodiniaceae enriched transcript sets in different treatments. Table S9. Functions of prokaryotic microbes enriched transcript sets in different treatments. Table S10. Detailed information of significantly correlated coral transcripts with Symbiodiniaceae physiology detected in GLMMLasso model. Table S11. Detailed information of significantly correlated prokaryotic transcripts with Symbiodiniaceae physiology detected in GLMMLasso model. Table S12. Parameters for analytical model example. Table S13. Nitrate concentration of each treatment during the 5-days experiment. Figure S1. MA plots of different comparisons (control-5, control-10, control-20, control-40) in coral (A, B, C, D), Symbiodiniaceae (E, F, G, H) and prokaryotic microbes (I, J, K, L). Figure S2. Prokaryotic community structures at phylum (A) and genus level (B) by Kraken2. Only genera with top 20 highest relative abundance were presented in the legend. Figure S3. Functions of shared transcripts in different comparisons. Representing coral (A), Symbiodiniaceae (B) and prokaryotic (C) shared transcripts, respectively. Figure S4. Functions of core transcripts in correlation networks. Representing coral (A) and Symbiodiniaceae (B) core transcripts in coral-algal network; Symbiodiniaceae (C) and prokaryotic microbial (D) core transcripts in algal-microbial network; coral (E) [file 40168_2023_1495_MOESM1_ESM.zip › Supplementary/FigS1.pdf]

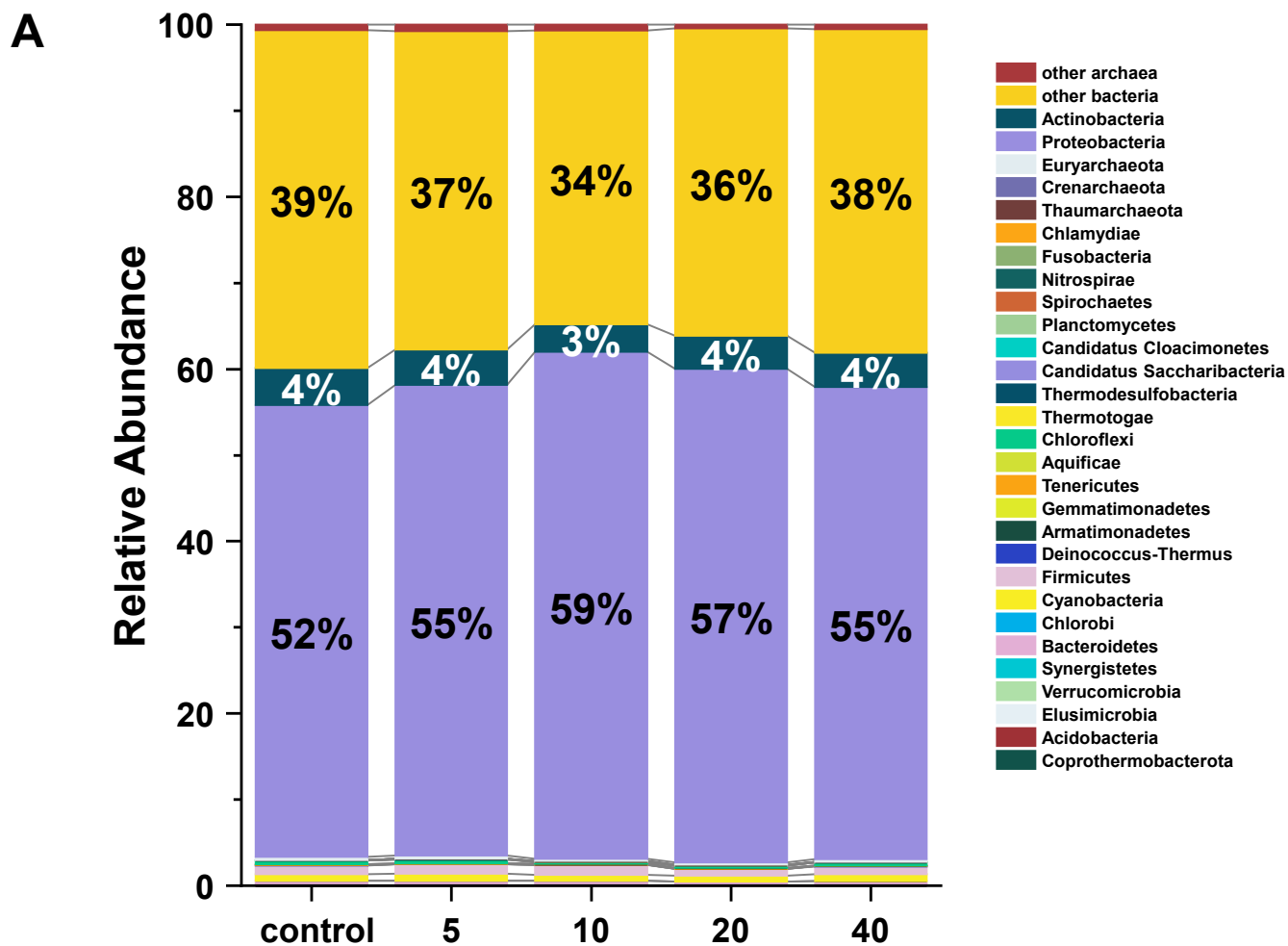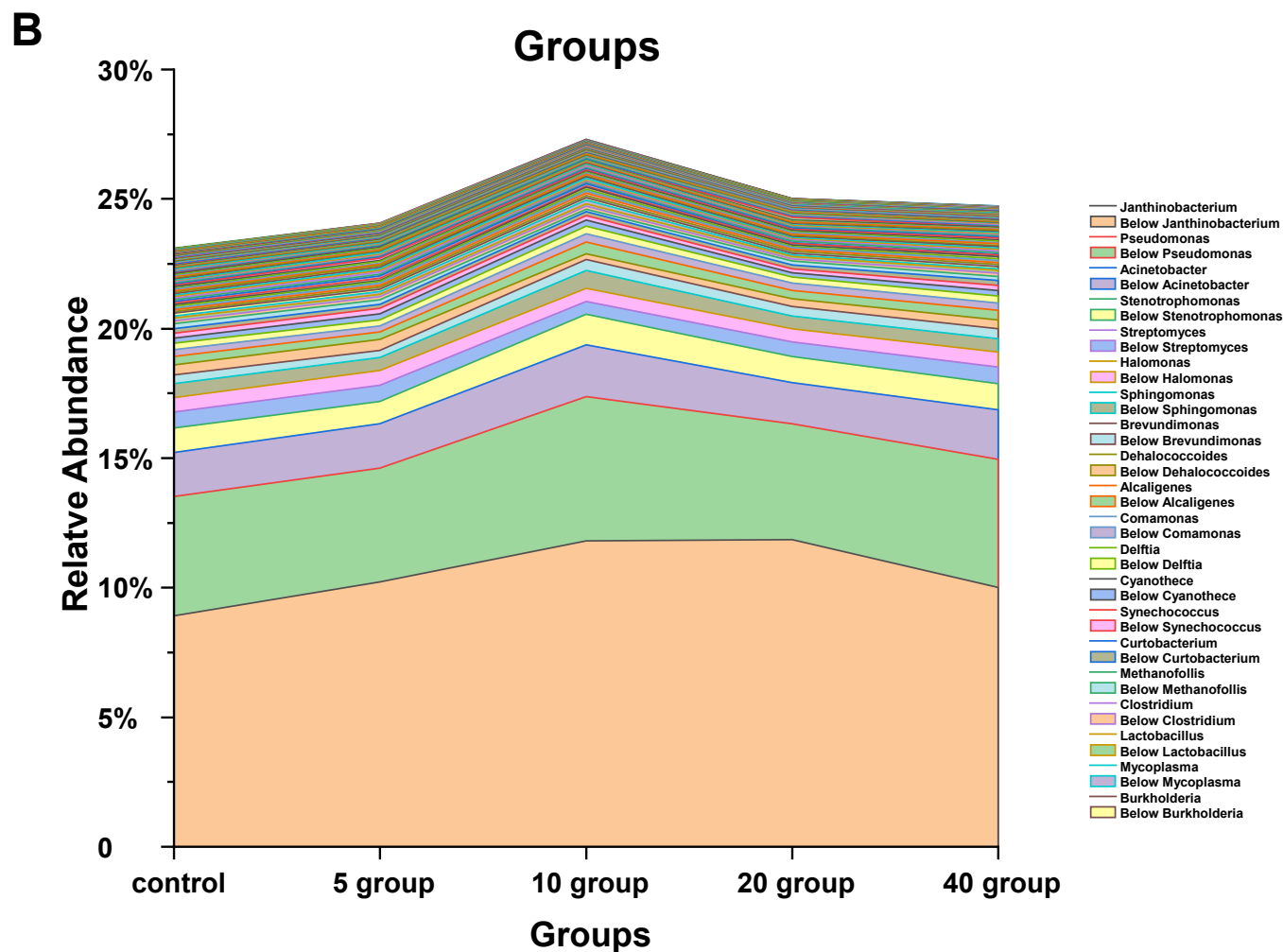

Supplement: Supplementary file 2 — Additional file 1: Table S1. Statistics of transcriptome de novo assembly. Table S2. Pair reads per sample mapping against different reference transcriptome. Table S3. Symbiodiniaceae community structure in different treatments. Table S4. Pathways related to coral differentially expressed transcripts in different treatments. Table S5. Pathways related to Symbiodiniaceae differentially expressed transcripts in different treatments. Table S6. Pathways related to prokaryotic differentially expressed transcripts in different treatments. Table S7. Functions of corals enriched transcript sets in different treatments. Table S8. Functions of Symbiodiniaceae enriched transcript sets in different treatments. Table S9. Functions of prokaryotic microbes enriched transcript sets in different treatments. Table S10. Detailed information of significantly correlated coral transcripts with Symbiodiniaceae physiology detected in GLMMLasso model. Table S11. Detailed information of significantly correlated prokaryotic transcripts with Symbiodiniaceae physiology detected in GLMMLasso model. Table S12. Parameters for analytical model example. Table S13. Nitrate concentration of each treatment during the 5-days experiment. Figure S1. MA plots of different comparisons (control-5, control-10, control-20, control-40) in coral (A, B, C, D), Symbiodiniaceae (E, F, G, H) and prokaryotic microbes (I, J, K, L). Figure S2. Prokaryotic community structures at phylum (A) and genus level (B) by Kraken2. Only genera with top 20 highest relative abundance were presented in the legend. Figure S3. Functions of shared transcripts in different comparisons. Representing coral (A), Symbiodiniaceae (B) and prokaryotic (C) shared transcripts, respectively. Figure S4. Functions of core transcripts in correlation networks. Representing coral (A) and Symbiodiniaceae (B) core transcripts in coral-algal network; Symbiodiniaceae (C) and prokaryotic microbial (D) core transcripts in algal-microbial network; coral (E) [file 40168_2023_1495_MOESM1_ESM.zip › Supplementary/FigS2.pdf]

**A**

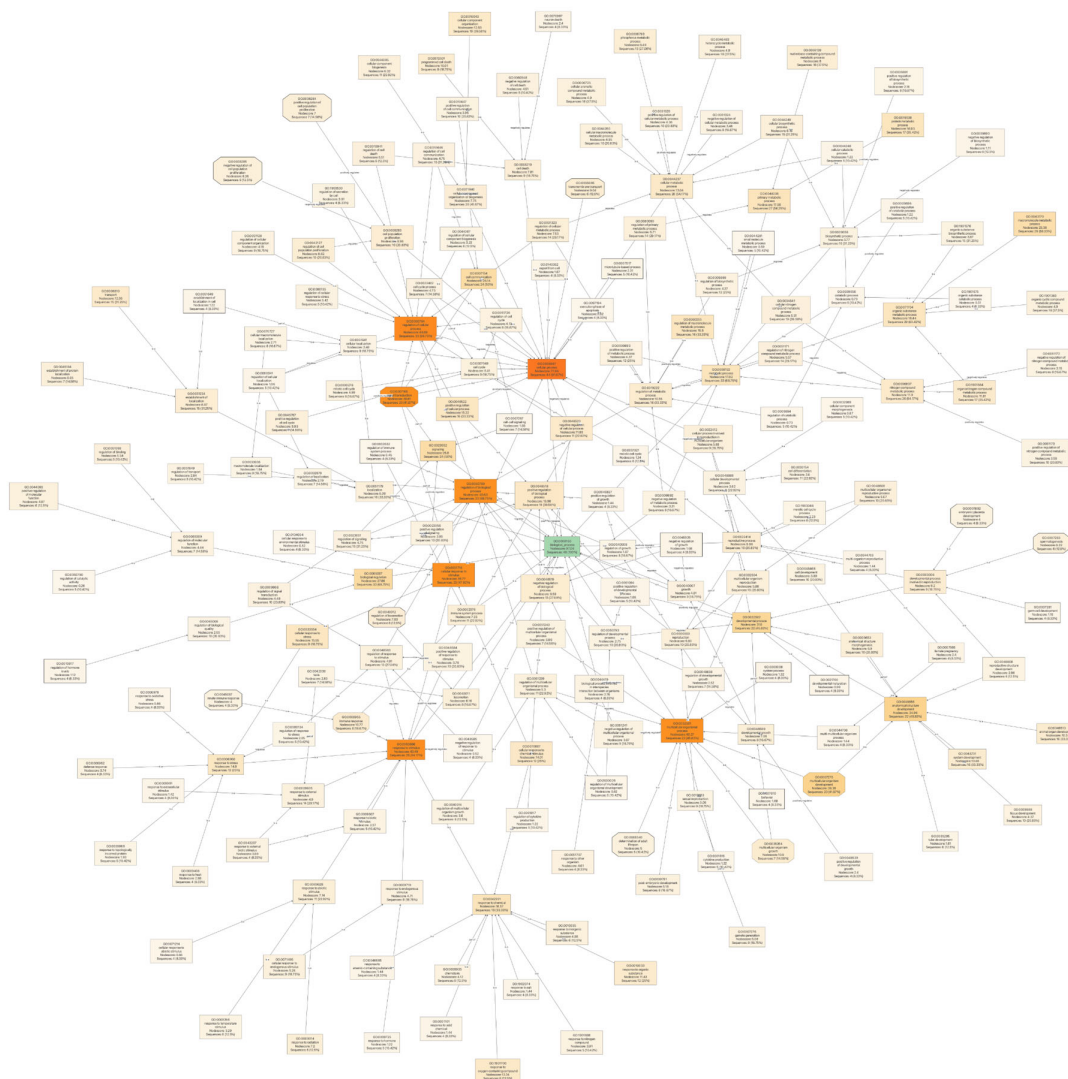

**B**

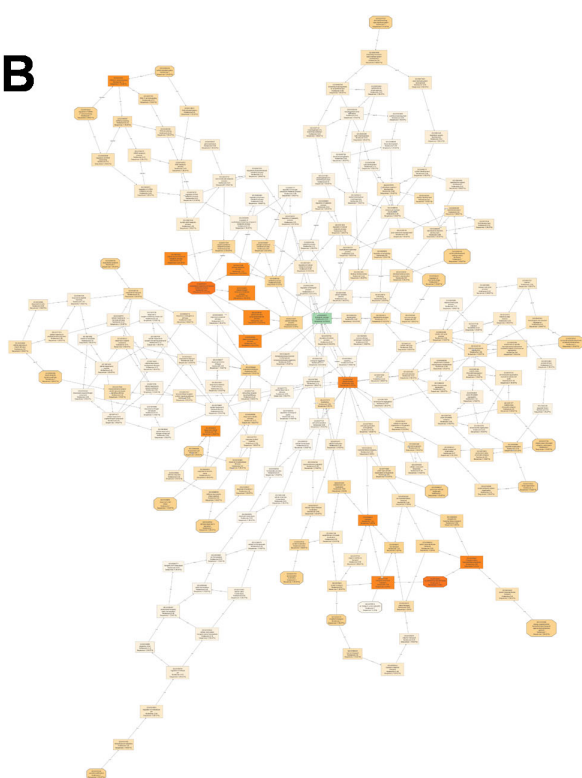

**C**

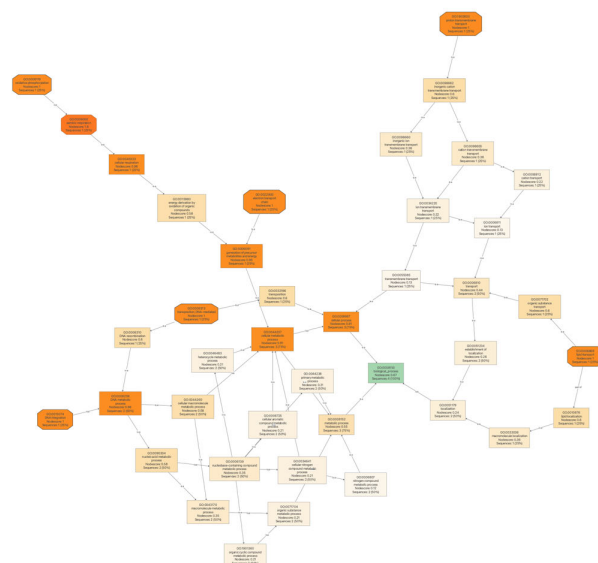

Supplement: Supplementary file 2 — Additional file 1: Table S1. Statistics of transcriptome de novo assembly. Table S2. Pair reads per sample mapping against different reference transcriptome. Table S3. Symbiodiniaceae community structure in different treatments. Table S4. Pathways related to coral differentially expressed transcripts in different treatments. Table S5. Pathways related to Symbiodiniaceae differentially expressed transcripts in different treatments. Table S6. Pathways related to prokaryotic differentially expressed transcripts in different treatments. Table S7. Functions of corals enriched transcript sets in different treatments. Table S8. Functions of Symbiodiniaceae enriched transcript sets in different treatments. Table S9. Functions of prokaryotic microbes enriched transcript sets in different treatments. Table S10. Detailed information of significantly correlated coral transcripts with Symbiodiniaceae physiology detected in GLMMLasso model. Table S11. Detailed information of significantly correlated prokaryotic transcripts with Symbiodiniaceae physiology detected in GLMMLasso model. Table S12. Parameters for analytical model example. Table S13. Nitrate concentration of each treatment during the 5-days experiment. Figure S1. MA plots of different comparisons (control-5, control-10, control-20, control-40) in coral (A, B, C, D), Symbiodiniaceae (E, F, G, H) and prokaryotic microbes (I, J, K, L). Figure S2. Prokaryotic community structures at phylum (A) and genus level (B) by Kraken2. Only genera with top 20 highest relative abundance were presented in the legend. Figure S3. Functions of shared transcripts in different comparisons. Representing coral (A), Symbiodiniaceae (B) and prokaryotic (C) shared transcripts, respectively. Figure S4. Functions of core transcripts in correlation networks. Representing coral (A) and Symbiodiniaceae (B) core transcripts in coral-algal network; Symbiodiniaceae (C) and prokaryotic microbial (D) core transcripts in algal-microbial network; coral (E) [file 40168_2023_1495_MOESM1_ESM.zip › Supplementary/FigS3.pdf]

A

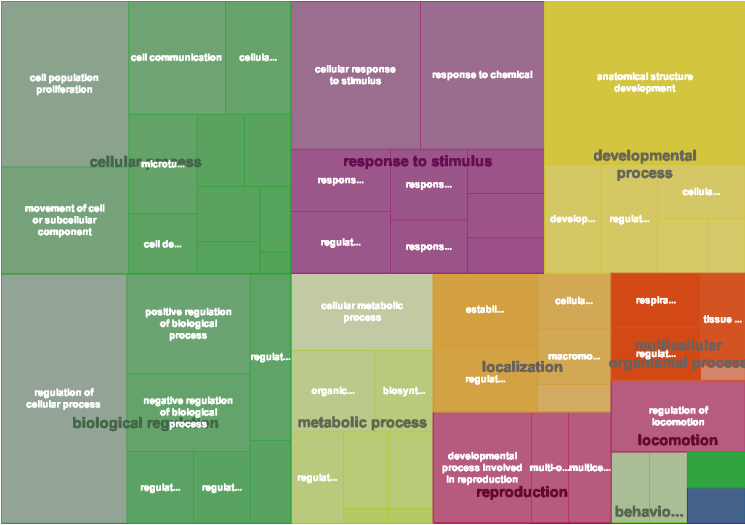

B

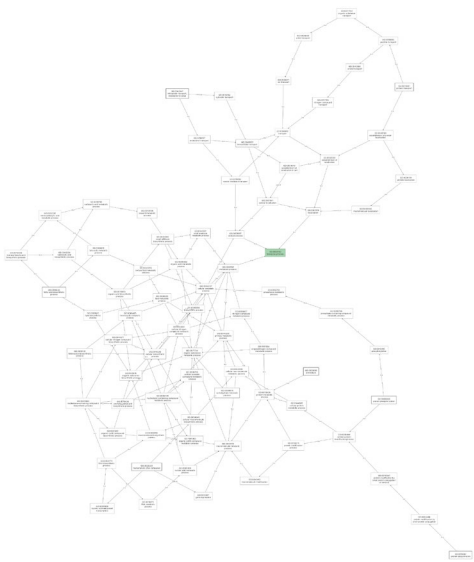

C

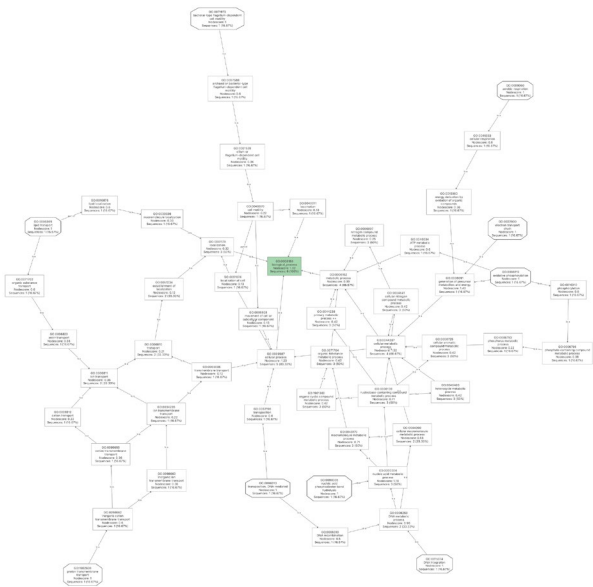

D

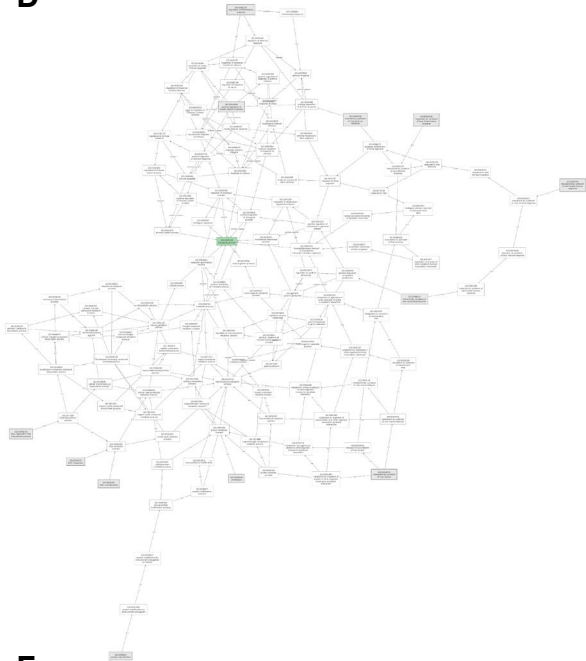

E

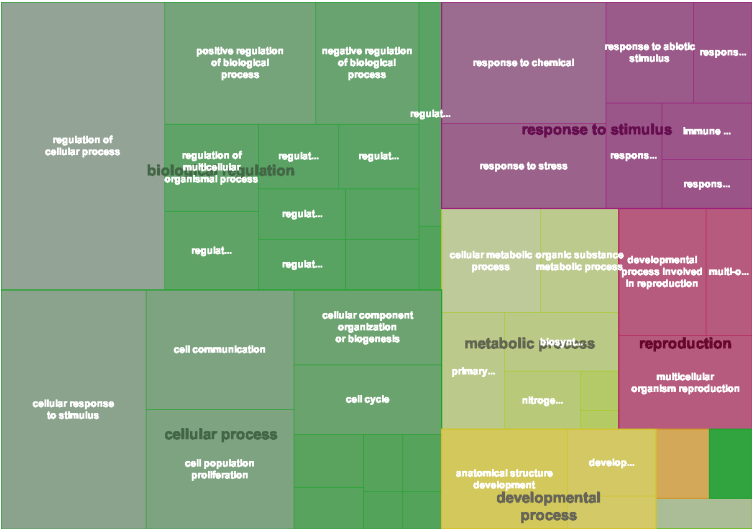

F

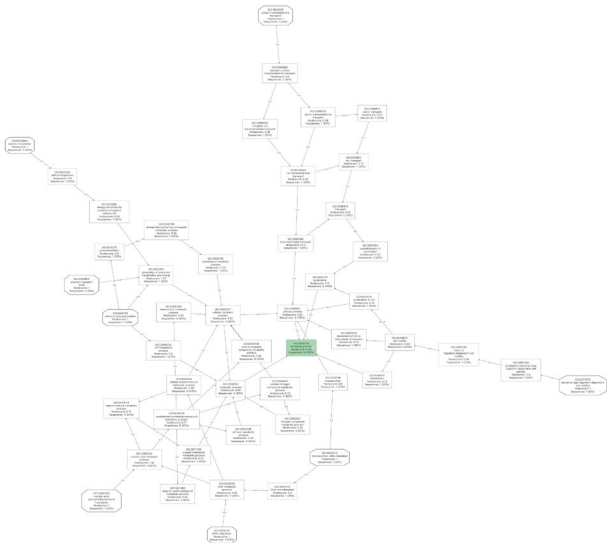

Supplement: Supplementary file 2 — Additional file 1: Table S1. Statistics of transcriptome de novo assembly. Table S2. Pair reads per sample mapping against different reference transcriptome. Table S3. Symbiodiniaceae community structure in different treatments. Table S4. Pathways related to coral differentially expressed transcripts in different treatments. Table S5. Pathways related to Symbiodiniaceae differentially expressed transcripts in different treatments. Table S6. Pathways related to prokaryotic differentially expressed transcripts in different treatments. Table S7. Functions of corals enriched transcript sets in different treatments. Table S8. Functions of Symbiodiniaceae enriched transcript sets in different treatments. Table S9. Functions of prokaryotic microbes enriched transcript sets in different treatments. Table S10. Detailed information of significantly correlated coral transcripts with Symbiodiniaceae physiology detected in GLMMLasso model. Table S11. Detailed information of significantly correlated prokaryotic transcripts with Symbiodiniaceae physiology detected in GLMMLasso model. Table S12. Parameters for analytical model example. Table S13. Nitrate concentration of each treatment during the 5-days experiment. Figure S1. MA plots of different comparisons (control-5, control-10, control-20, control-40) in coral (A, B, C, D), Symbiodiniaceae (E, F, G, H) and prokaryotic microbes (I, J, K, L). Figure S2. Prokaryotic community structures at phylum (A) and genus level (B) by Kraken2. Only genera with top 20 highest relative abundance were presented in the legend. Figure S3. Functions of shared transcripts in different comparisons. Representing coral (A), Symbiodiniaceae (B) and prokaryotic (C) shared transcripts, respectively. Figure S4. Functions of core transcripts in correlation networks. Representing coral (A) and Symbiodiniaceae (B) core transcripts in coral-algal network; Symbiodiniaceae (C) and prokaryotic microbial (D) core transcripts in algal-microbial network; coral (E) [file 40168_2023_1495_MOESM1_ESM.zip › Supplementary/FigS4.pdf]

**A**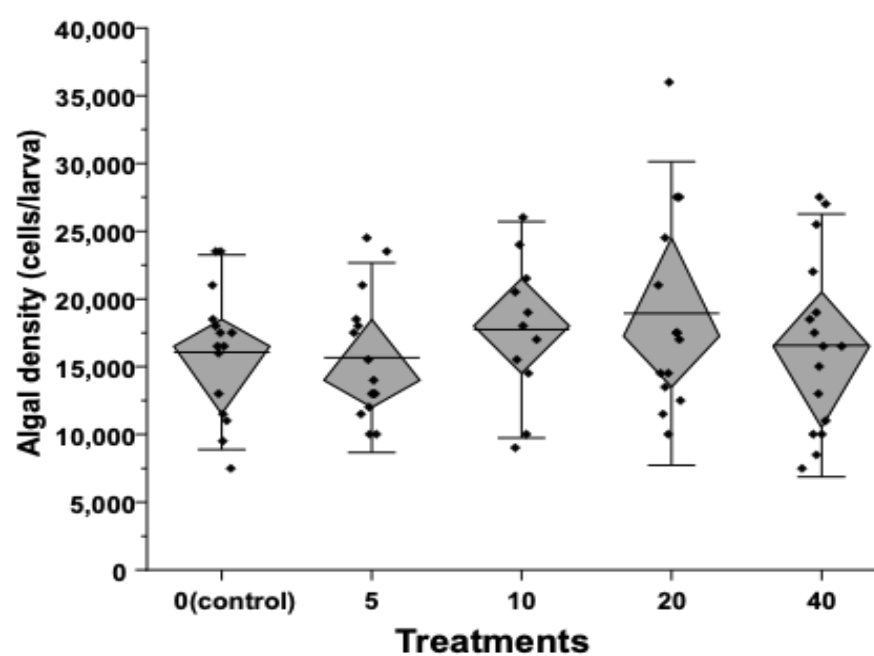**B**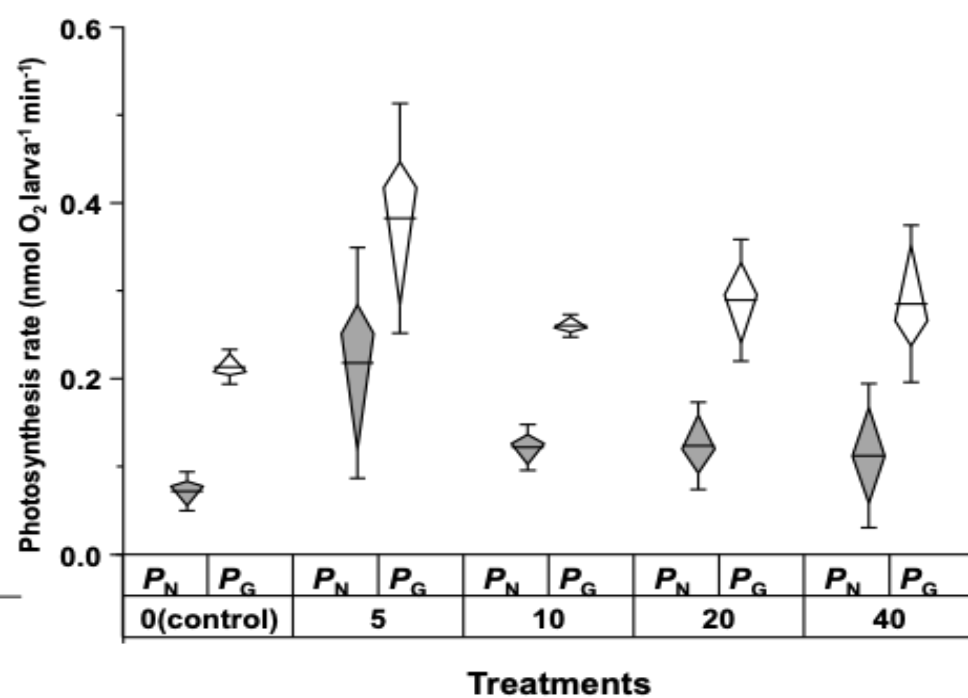

Supplement: Supplementary file 2 — Additional file 1: Table S1. Statistics of transcriptome de novo assembly. Table S2. Pair reads per sample mapping against different reference transcriptome. Table S3. Symbiodiniaceae community structure in different treatments. Table S4. Pathways related to coral differentially expressed transcripts in different treatments. Table S5. Pathways related to Symbiodiniaceae differentially expressed transcripts in different treatments. Table S6. Pathways related to prokaryotic differentially expressed transcripts in different treatments. Table S7. Functions of corals enriched transcript sets in different treatments. Table S8. Functions of Symbiodiniaceae enriched transcript sets in different treatments. Table S9. Functions of prokaryotic microbes enriched transcript sets in different treatments. Table S10. Detailed information of significantly correlated coral transcripts with Symbiodiniaceae physiology detected in GLMMLasso model. Table S11. Detailed information of significantly correlated prokaryotic transcripts with Symbiodiniaceae physiology detected in GLMMLasso model. Table S12. Parameters for analytical model example. Table S13. Nitrate concentration of each treatment during the 5-days experiment. Figure S1. MA plots of different comparisons (control-5, control-10, control-20, control-40) in coral (A, B, C, D), Symbiodiniaceae (E, F, G, H) and prokaryotic microbes (I, J, K, L). Figure S2. Prokaryotic community structures at phylum (A) and genus level (B) by Kraken2. Only genera with top 20 highest relative abundance were presented in the legend. Figure S3. Functions of shared transcripts in different comparisons. Representing coral (A), Symbiodiniaceae (B) and prokaryotic (C) shared transcripts, respectively. Figure S4. Functions of core transcripts in correlation networks. Representing coral (A) and Symbiodiniaceae (B) core transcripts in coral-algal network; Symbiodiniaceae (C) and prokaryotic microbial (D) core transcripts in algal-microbial network; coral (E) [file 40168_2023_1495_MOESM1_ESM.zip › Supplementary/FigS5.pdf]

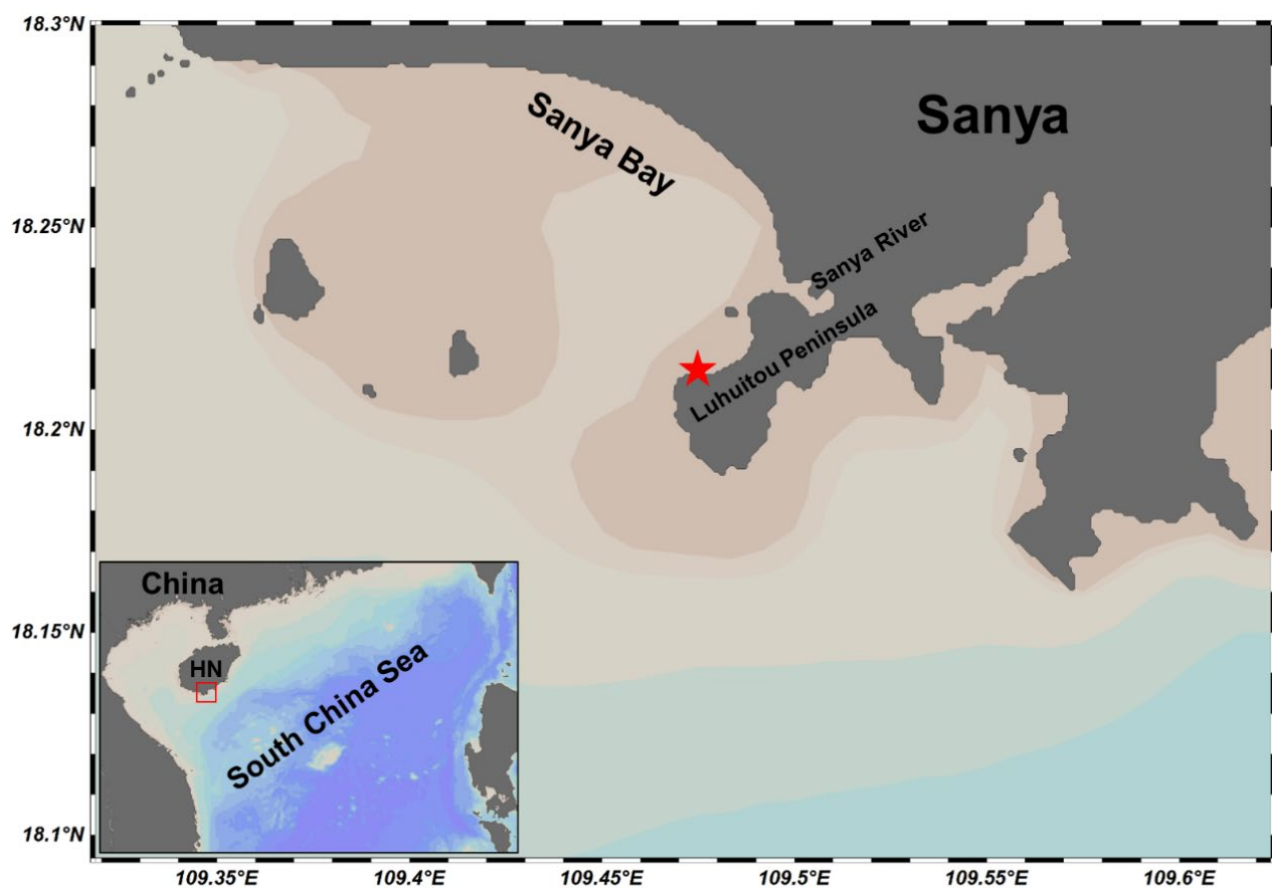

Supplement: Supplementary file 2 — Additional file 1: Table S1. Statistics of transcriptome de novo assembly. Table S2. Pair reads per sample mapping against different reference transcriptome. Table S3. Symbiodiniaceae community structure in different treatments. Table S4. Pathways related to coral differentially expressed transcripts in different treatments. Table S5. Pathways related to Symbiodiniaceae differentially expressed transcripts in different treatments. Table S6. Pathways related to prokaryotic differentially expressed transcripts in different treatments. Table S7. Functions of corals enriched transcript sets in different treatments. Table S8. Functions of Symbiodiniaceae enriched transcript sets in different treatments. Table S9. Functions of prokaryotic microbes enriched transcript sets in different treatments. Table S10. Detailed information of significantly correlated coral transcripts with Symbiodiniaceae physiology detected in GLMMLasso model. Table S11. Detailed information of significantly correlated prokaryotic transcripts with Symbiodiniaceae physiology detected in GLMMLasso model. Table S12. Parameters for analytical model example. Table S13. Nitrate concentration of each treatment during the 5-days experiment. Figure S1. MA plots of different comparisons (control-5, control-10, control-20, control-40) in coral (A, B, C, D), Symbiodiniaceae (E, F, G, H) and prokaryotic microbes (I, J, K, L). Figure S2. Prokaryotic community structures at phylum (A) and genus level (B) by Kraken2. Only genera with top 20 highest relative abundance were presented in the legend. Figure S3. Functions of shared transcripts in different comparisons. Representing coral (A), Symbiodiniaceae (B) and prokaryotic (C) shared transcripts, respectively. Figure S4. Functions of core transcripts in correlation networks. Representing coral (A) and Symbiodiniaceae (B) core transcripts in coral-algal network; Symbiodiniaceae (C) and prokaryotic microbial (D) core transcripts in algal-microbial network; coral (E) [file 40168_2023_1495_MOESM1_ESM.zip › Supplementary/FigS6.pdf]
